# Is the toxic potential of nanosilver dependent on its size?

**DOI:** 10.1186/s12989-014-0065-1

**Published:** 2014-12-03

**Authors:** Anna Huk, Emilia Izak-Nau, Bogumila Reidy, Matthew Boyles, Albert Duschl, Iseult Lynch, Maria Dušinska

**Affiliations:** Department of Environmental Chemistry, Health Effects Laboratory, NILU, Instituttveien 18, 2007 Kjeller, Norway; Department of Molecular Biology, University of Salzburg, Salzburg, Austria; Bayer Technology Services GmbH, Leverkusen, Germany; Centre for BioNano Interactions, School of Chemistry and Chemical Biology University College Dublin, Belfield, Dublin, 4 Ireland; School of Geography, Earth & Environmental Sciences, University of Birmingham, Edgbaston, B15 2TT, Birmingham, UK

**Keywords:** Size-related nanomaterial toxicity, Silver nanomaterials, Uptake and localisation, Cytotoxicity, Inflammation, DNA damage, Mutagenicity

## Abstract

**Background:**

Nanosilver is one of the most commonly used engineered nanomaterials (ENMs). In our study we focused on assessing the size-dependence of the toxicity of nanosilver (Ag ENMs), utilising materials of three sizes (50, 80 and 200 nm) synthesized by the same method, with the same chemical composition, charge and coating.

**Methods:**

Uptake and localisation (by Transmission Electron Microscopy), cell proliferation (Relative growth activity) and cytotoxic effects (Plating efficiency), inflammatory response (induction of IL-8 and MCP-1 by Enzyme linked immune sorbent assay), DNA damage (strand breaks and oxidised DNA lesions by the Comet assay) were all assessed in human lung carcinoma epithelial cells (A549), and the mutagenic potential of ENMs (Mammalian *hprt* gene mutation test) was assessed in V79-4 cells as per the OECD protocol. Detailed physico-chemical characterization of the ENMs was performed in water and in biological media as a prerequisite to assessment of their impacts on cells. To study the relationship between the surface area of the ENMs and the number of ENMs with the biological response observed, Ag ENMs concentrations were recalculated from μg/cm^2^ to ENMs cm^2^/cm^2^ and ENMs/cm^2^.

**Results:**

Studied Ag ENMs are cytotoxic and cytostatic, and induced strand breaks, DNA oxidation, inflammation and gene mutations. Results expressed in mass unit [μg/cm^2^] suggested that the toxicity of Ag ENMs is size dependent with 50 nm being most toxic. However, re-calculation of Ag ENMs concentrations from mass unit to surface area and number of ENMs per cm^2^ highlighted that 200 nm Ag ENMs, are the most toxic. Results from *hprt* gene mutation assay showed that Ag ENMs 200 nm are the most mutagenic irrespective of the concentration unit expressed.

**Conclusion:**

We found that the toxicity of Ag ENMs is not always size dependent. Strong cytotoxic and genotoxic effects were observed in cells exposed to Ag ENMs 50 nm, but Ag ENMs 200 nm had the most mutagenic potential. Additionally, we showed that expression of concentrations of ENMs in mass units is not representative. Number of ENMs or surface area of ENMs (per cm^2^) seem more precise units with which to compare the toxicity of different ENMs.

**Electronic supplementary material:**

The online version of this article (doi:10.1186/s12989-014-0065-1) contains supplementary material, which is available to authorized users.

## Background

Nanosilver is one of the most commonly used engineered nanomaterials (ENMs), and because of its unique physico-chemical properties and antibacterial potential it plays an important role in many industries, including food packaging, textiles production, agriculture and water disinfection [1-5]. Nevertheless, the commercially most important applications are in medicine where it is used in bioimaging, biosensors, dental products, implants and wound dressings [6-8]. Medical products with nanosilver (Ag ENMs) were registered more than 60 years ago, however, the toxic potential of this ENM is still not well understood with significant debate as to whether the toxicity is entirely related to the dissolved ion fraction or can result from the nanoform also [9]. The first toxicology studies have reported controversial results. It has been shown that Ag ENMs can induce oxidative stress in human liver cells, DNA damage in testicular cells, human fibroblasts and peripheral blood cells, including DNA oxidation in human kidney cells and apoptosis in HeLa and human liver cells [10-15]. In other studies, Ag ENMs did not cause toxic effects in bone marrow cells, erythrocytes or human keratinocytes [16,17]. These discrepancies in toxicology studies may be explained by cell-type specific responses or coupled with the huge differences in the properties of the Ag ENMs used [18]. One of the main reasons for discrepancies between results in nanotoxicology studies *in vitro* is the lack of characterization of ENMs, especially under the actual exposure conditions in the relevant culture media. This includes also a lack of information about biological and chemical contamination of ENMs in the samples, and differences in experimental conditions such as in amount of protein in the cell culture media and whether the serum was heat inactivated or not [19-23]. In addition, a large part of these discrepancies is likely due to the fact that the ionic form (Ag^+^) is often present in an stock solutions in parallel to nanoforms due to its high dissolution potential [24]. The presence of Ag ions in the Ag ENM stocks can vary depending on ENMs preparation protocol or storage conditions [Izak-Nau E, Huk A, Reidy B, Uggerud H, Vadset M, Eiden S, Voetz M, Duschl A, Dušinska M, Lynch I: Impact of Storage Conditions and Storage Time on Silver Nanoparticle Physicochemcial Properties and Implications for Biological Effects. Manuscript in preparation].

The aim of the present study is a nanotoxicology evaluation of high-quality stable Ag ENMs, with 3 different sizes (50, 80 and 200 nm), coated by polyvinylpyrrolidone (PVP) [25], prepared to ensure absence of Ag ions. Detailed characterization of the ENMs was performed to measure parameters which may influence the uptake of ENMs by cells and the biological response.

Toxic effects of Ag ENMs on lung cells were investigated previously [26-28]. However, no study has investigated the mutagenic effect of Ag ENMs using the Mammalian *hprt* gene mutation assay. Most studies on size dependent toxicity expressed the ENM concentration in mass units. In our research, Ag ENM concentrations were additionally re-calculated from mass units [μg/cm^2^] to surface area of ENMs [ENMs cm^2^/cm^2^] and number of ENMs [ENMs/cm^2^] as for nanoforms these units are suggested to be more relevant concentration descriptors.

A large number of consumer products which can generate aerosolised Ag are already on the market including haircare products, antibacterial sprays, or air conditioning cleaning products. Ag in aerosol form can reach the human body by different routes; one of the main routes is inhalation where the principle targets are lung cells, which is the biological *in vitro* model considered in our study.

A human type II alveolar epithelial lung cell line (A549) was selected, since it is a common model for toxicity studies representing the lung, the major target organ for ENM accumulation by inhalation exposure [29]. Additionally, for the mutation study of ENM, we used the *hprt* gene mutation assay on Chinese hamster lung fibroblast cells (V79-4), according to the test guideline OECD 476 [30,31].

All used materials were synthesized by the same method, with minimum differences between batches, fully characterized by standard techniques: ENM size distribution and aggregation/agglomeration by dynamic light scattering (DLS), transmission and scanning electron microscopy (TEM and SEM) and analytical ultracentrifugation (AC); chemical composition by X-ray photoelectron spectroscopy (XPS), secondary ion mass spectroscopy and X-ray diffraction (XRD); surface composition by XPS and time-of-flight secondary ion mass spectrometry IV (ToF-SIMS); surface charge by zeta potential and specific surface area by the Brunauer-Emmett-Teller method (BET). For the toxicity studies, a range of different endpoints were addressed and standard methods have been applied: cell proliferation and cytotoxicity (Plating efficiency (PE) and Relative growth activity (RGA); genotoxicity (Comet assay); release of inflammatory markers (Enzyme-linked immunosorbent assay; ELISA) and mutagenicity (Mammalian *in vitro hprt* gene mutation tests, OECD 476). Uptake and subcellular localization of the ENMs was studied using TEM. Additionally, to study the relationship between surface area of ENMs and number of ENMs and the observed biological responses, all toxicology results were expressed as mass units, surface area of ENMs and number of ENMs (per cm^2^).

## Methods

### Ag ENM synthesis

To synthesize Ag ENMs, 1 g of Luvitec K90® (PVP) was dissolved in 50 ml of 0.054 M NaOH aqueous solution. The reaction solution was poured directly into 0.054 M AgNO_3_ and stirred at 400 rpm (50 nm: t = 60 min, T = 21°C, 80 nm: t = 60 min, T = 60°C, 200 nm: t = 30 min, T = 80°C). Ten ml of formaldehyde 37% aqueous solution (FA) was added to the reaction mixture with stirring for 1 h at RT.

After the reaction times the solutions were transferred onto ice for 1 h. The ENM dispersions were centrifuged at 15000 rpm (50 nm), 12000 rpm (80 nm) and 8000 rpm (200 nm) for 20 min. The ENMs were washed with acetone, and then with deionized water, to remove any unreacted FA and PVP. Subsequently, the ENM dispersions were sonicated with a 5 mm microtip for 5 min at 30% amplitude (Sonikator-Digital Sonifer® 450; Branson Ultrasonics Corporation). The concentration of the ENMs was determined to be 1% using a Halogen Moisture Analyzer (Mettler Toledo, HR73).

### Cell lines

Human lung carcinoma epithelial cells (A549) were cultured in flasks in RPMI 1640 media (Sigma) with 10% heat inactivated foetal bovine serum (FBS; Sigma), 1% penicillin – streptomycin (Sigma) in a humidified atmosphere of 5% CO_2_ and 37°C. In each experiment cells of passages between 3–6 were used.

Chinese hamster lung fibroblast cells (V79-4) were cultured in flasks in DMEN low glucose media (Sigma), with activated 10% FBS, 1% penicillin–streptomycin and L-glutamine (Sigma) in a humidified atmosphere of 5% CO_2_ at 37°C.

### Physicochemical characterization

The hydrodynamic size/size distribution and zeta potential of the pristine ENMs were measured using a Zetasizer 3000 HSa, Malvern Instruments. The ENMs size was assessed by DLS using a He-Ne laser (633 nm) as the light source. The stock suspension was diluted with deionized water to result in a count rate of 100–500 kcps. ENM sizing measurements were performed in 10 mm polystyrene cuvettes at 25°C. The results are given as Z-average values of the number, volume and intensity size distributions. The zeta potential was determined by Laser Doppler Electrophoresis (LDE) using a quartz capillary electrophoresis cell. All measurements were performed in triplicate for a single batch of ENMs and the results were the average of the three measurements.

The ENM size distributions were additionally determined by AC using a Beckman Ultracentrifuge type XL70, equipped with an optical device. A diode laser (695 nm) with an optical fiber was used as the light source. It was operated at the constant voltage of 6 V using a T4N16B8 generator from Gossen. A 3 mm Beckman quartz cell was used as the ultracentrifuge cell with a gap width of about 0.3 mm for the passage of the light. The samples were diluted with deionized water to obtain concentrations ranging from 0.5-0.05%. Depending on the ENM sizes, the samples were centrifuged for 10–120 min at speeds between 4000 and 50000 U/min.

The primary ENM sizes and shapes were assessed using a Phillips CM20 TEM working at 200 keV. For TEM analysis, stock ENM suspensions were diluted 1:100 in deionized water and 3 μl was pipetted onto holey carbon grids (S162, Plano GmbH) and subsequently left to evaporate. Around 700 ENMs were selected to estimate ENMs size/size distribution using the analysis pro software.

The primary ENM sizes and shapes were additionally assessed using an FEI Sirion 100 T SEM working at 10 keV. For SEM analysis, 20 μl stock suspensions were dried directly onto the carbon adhesive pad of an SEM sample holder.

The chemical and elemental composition of the ENMs were examined with a PHI VersaProbe 5000 XPS, using a monochromated Al Kα X-ray beam scanned over 600 μm × 400 μm area (200 μm diameter/50 W X-ray beam) or 1400 μm × 100 μm (100 μm diameter/100 W X-ray beam) at a fixed take-off angle of 45°. For XPS analysis, the stock suspensions were dried onto an indium or silicon surface. Spectra evaluation of 10 total measuring cycles was performed using MultiPack-Version 9.2 software. The results in % were derived from the relative concentrations of elements and their chemical bonds from line shape analysis.

Surface chemistry measurements were performed using a ToF-SIMS. The primary ion species used was 10 keV Ga^+^, scanning an area of typically 150 × 150 μm^2^. For SIMS analysis, the stock suspensions were dried onto a silicon surface.

Crystallite size and crystalline phase were evaluated by XRD using a PANalytical EMPYREAN PIXcel with 3D Counter, operating at a voltage of 40 kV and a current of 40 mA with Cu Kα and Kβ radiation. For XRD analysis, the stock suspensions were dried onto a silicon surface.

Specific surface area was determined using the BET method, from nitrogen adsorption/desorption isotherms, recorded at 77 K on a Gemini 2360 from Micromeritics S/N 3014. The measuring range was 0.1-1000 m^2^/g. The stock solution was freeze dried to obtain 0.5 g of the examined sample. ENM concentration was additionally analyzed with a Halogen Moisture Analyzer (Mettler Toledo, HR73). One gram of the stock solution was placed onto the analyzer plate and left to allow solvent evaporation to give the wt/wt % value.

To investigate the ENM stability in biological media, the ENM dispersions were prepared in DMEM or RPMI 1600 medium, supplemented with 10% FBS. The ENMs were incubated for 2, 24 and 48 h (relevant to the toxicological tests) at 37°C in a CO_2_ incubator. The ENM properties in the biological environment were characterized using DLS.

A previous study utilising these Ag ENMs assessed the kinetics of dissolution and found that even after 4 months of storage (in water, t = 5°C) no significant changes in Ag ENMs physico-chemical properties, including dissolution, were observed [32]. While not explicitly assessed, no significant dissolution in cell culture media is expected over the timeframe of the studies, as a result of the PVP capping, which reduces dissolution [33] and reduces protein binding [34]. The ENMs were produced and handled under semi-sterile conditions, and no endotoxin contamination was found during random checking of ENMs (performed by Yang Li, Nazionale delle Institute de la Ricerche, Pisa, Italy as part of the EU FP7 NanoTOES consortium) using the LAL assay (personal communication, and publication in preparation).

### Uptake and localisation of ENMs - transmission electron microscopy (TEM)

A549 cells were grown on 35 mm Petri dishes, at a density which allowed them to reach 80% confluence at the end of the exposure period (2, 24 and 48 h). 24 h after seeding, the cells were exposed to Ag ENMs 50 nm (5.3 μg/cm^2^, 2.7 × 10^11^ ENMs/cm^2^), 80 nm (5.3 μg/cm^2^, 0.55 × 10^11^ ENMs/cm^2^) and 200 nm (10.5 μg/cm^2^, 0.2 × 10^11^ ENMs/cm^2^) for 2, 24 and 48 h. After exposure, cells were fixed in 2.5% glutaraldehyde in 0.1 M Sorensen phosphate buffer (pH 7.3) for at least 1 h. Afterwards, cells were washed with 0.1 M Sorensen phosphate buffer (pH 7.3) and post-fixed in 1% osmium tetroxide in deionised water for 1 h. Samples were then dehydrated in increasing concentrations of ethanol (from 70% to 100%, 10–20 min each step), immersed in ethanol/Epon (1:1 v/v) mixture and embedded in pure Epon (2 h in 37°C) and polymerised for 24 h at 60°C. Sections (~80 nm) were cut using a diamond knife on an ultra-microtome Leica EM UC6 and mounted on copper grids. Before image acquisition, sections were stained using uranyl acetate and lead citrate. All images were acquired on an FEI TECNAI 120 TEM (120 kV).

### Proliferation assay - relative cell growth activity (RGA)

A549 cells were seeded on 6-well plates (1.2 × 10^5^ cells per well) and incubated at 37°C. After 24 h, the cells were exposed to Ag ENMs (50, 80 or 200 nm) for 2, 24 and 48 h at concentrations ranging from 1.1-21.1 μg/cm^2^. At the end of the exposure period, medium was removed; cells were washed with PBS, trypsinized and re-suspended in 1 ml medium. 10 μl of the cell suspension was mixed with 10 μl 0.4% trypan blue (Invitrogen) and the percentages of living cells (unstained) and stained cells with damaged membranes were measured using a Countess™ Automated Cell Counter (Invitrogen). Measurements were performed immediately upon staining (for all three exposure times).

RGA was calculated according to the following formula:

$$ RGA\ \left(\%\right) = \frac{\left( number\  of\  living\  cells\  at\  day\ n/ number\  of\  seeded\  cells\  at\  day\ 0\right)\  in\  exposed\  cultures\ }{\left( number\  of\  living\  cells\  at\  day\ n/ number\  of\  seeded\  cells\  at\  day\ 0\right)\  in\  unexposed\  cultures}\times 100\ \% $$

### Plating efficiency (PE)

A549 cells were exposed to Ag ENMs, washed and counted as described above. 100 cells per well were inoculated in 6-well plates (1 plate for each ENM size/tested concentration) and left in an incubator at 37°C for 10 days. The cells were then stained with 1% methylene blue (Sigma) and the number of colonies was counted manually.

PE was calculated according to the follow formula:

$$ PE\ \left(\%\right)=\frac{number\  of\  colonies\  in\  exposed\  cultures}{number\  of\  colonies\  in\  unexposed\  cultures}\times 100\ \% $$

### Cell morphology

To observe changes in cell morphology such as rounded, shrunken or detached cells, A549 cells were seeded in 6-well cell culture plates at a density of 1.2 × 10^5^ cells/ml. The next day, cells were treated with Ag ENMs (21.2 μg/cm^2^, 50, 80 or 200 nm) for 24 h and observed under an optical microscope (Leica, model DM-IL). Images were captured under 100× magnification using the microscope’s camera (Motic, model Motican 3 software Motic Images 2.0 ML). Cell morphology was analysed visually by comparing about 90 images each of untreated and treated cells.

### Enzyme-linked immunosorbent assay (ELISA)

The ELISA assay, for IL-8 and MCP-1, was used to assess the immune response of A549 cells exposed to Ag ENMs (50, 80 or 200 nm). A549 cells were seeded on 24-well plates (0.25 × 10^5^ cells per well) and incubated at 37°C. Cells were exposed to Ag ENMs for 24 h at concentrations ranging from 0.21-15.6 μg/cm^2^. After exposure, supernatant was collected and centrifuged at 14000 rpm for 5 min. A 96 well plate was pre-coated with specific capture antibodies (0.5 μg/ml) (Peprotech; NJ, USA) and left overnight at 4°C. The following day the plate was washed 3 times (PBS, 0.05% Tween) and 100 μl blocking buffer (PBS, 1% BSA) was added to each well and incubated for 2 h at room temperature in the dark and then the plate was washed again. Exposure medium, blank and protein standards (15.6–1000 pg/ml) were placed in wells and were incubated for 2 h. The plate was then washed and incubated with detection antibodies (0.5 μg/ml) (Peprotech) for 1 h, then washed again and incubated with avidin peroxidase conjugate for 30 min. Again the plate was washed and 3,3’,5,5 tetramethyl benzidine liquid substrate (Sigma) was added. The reaction was stopped using 2 M H_2_SO_4_ (Sigma). To analyse the concentration of inflammatory markers present, the absorbance at 450 nm was measured using a Tecan Plate Reader. As a positive control, cells were treated with TNFα (20 ng/ml, 24 h) (ImmunoTools; Friesoythe, Germany).

### The Comet assay

The Comet assay was used to assess the genotoxicity of the Ag ENMs. Glass microscope slides were precoated with melted 0.5% normal melting point (NMP) agarose (Sigma) and let dry for at least 24 h.

A549 cells were exposed to Ag ENMs, washed and counted as described above. After exposure of cells, medium was removed, cells were washed with PBS, trypsinized and re-suspended in 1 ml of medium. Cell suspensions (10^4^ cells) were re-suspended in 200 μl 1% low melting point agarose (LMP, Sigma). 10 μl of mixture was dropped onto pre-coated glass slides (2 drops per concentration, 12 drops per slide) and placed in the fridge for 10 min. Slides were then put into lysis solution (2.5 M NaCl, 0.1 M EDTA, 10 mM Tris, 10% Triton X-100) [31]. After lysis, slides were subjected to alkaline solution (0.3 M NaOH, 1 mM EDTA) for DNA unwinding, followed by electrophoresis at 25 V for 20 min in a standard Comet assay electrophoresis tank. Slides were then washed in PBS and subsequently in water and left overnight to dry. Slides were stained with SybrGold (0.1 μl of stock per 1 ml of TE buffer - 10 mM TrisHCl, 1 mM Na_2_EDTA, pH 7.5-8, Invitrogen), covered with a cover slip and examined by fluorescence microscopy (Leica DMI 6000 B). Images of comets were scored using image analysis Comet Assay IV software (Perspective Instruments), calculating the median of % DNA in the tail from 50 comets per gel and the mean from 3 experiments.

For detection of oxidative DNA damage we used the modified version of the Comet assay with formamidopyrimidine DNA glycosylase (FPG). The FPG enzyme was kindly provided by Professor Andrew Collins (Department of Nutrition, University of Oslo, Norway). After lysis, the slides were washed with FPG buffer (40 mM HEPES, 0.1 M KCl, 0.5 mM EDTA, 0.2 mg/ml bovine serum albumin, pH 8.0) and then incubated with FPG enzyme (30 μl/gel, 30 min, 37°C and a humidified atmosphere). Further steps were performed according to the standard Comet assay protocol described above. DNA oxidation lesions (NET FPG) were calculated as the difference between % DNA in the tail in samples with FPG enzyme treatment and % DNA in the tail in samples without FPG enzyme treatment. Positive controls were hydrogen peroxide (Sigma) (50 μM, 5 min, on ice) for strand breaks (SBs) and the photosensitiser Ro19-8022 (Hoffman La Roche) plus visible light (1 μM in PBS, 5 min, on ice) for DNA oxidation (NET FPG).

### Mammalian *in vitro hprt* gene mutation test (OECD 476)

V79-4 cells were seeded on 6-well plates (1 × 10^5^ cells per well) and incubated at 37°C. After 24 h, the cells were exposed to Ag ENMs (50, 80, 200 nm) for 24 h, at concentrations ranging from 0.21–15.6 μg/cm^2^. After exposure, the medium was removed, and cells were washed, trypsinized and re-suspended in 2 ml medium. Cells were seeded in ϕ100 mm Petri dishes (3.5 × 10^5^ cells/Petri dish, 3 dishes per sample to achieve 10^6^ cells per sample), and cultivated in culture medium for an additional 8 days. Cells were harvested twice for mutations at days 6 and 8 after the treatment: cells were inoculated in ϕ100 mm Petri dishes (2 × 10^5^ cells/Petri dish, 5 dishes per sample to achieve 10^6^ cells per sample) and grown in selective medium containing 6-thioguanine (5 μg/ml, Sigma) for 10 days to form colonies. Mutant colonies were stained with 1% methylene blue and counted manually. Only colonies with a minimum of 50 cells were counted.

The number of surviving cells was assessed by PE assay. On days 0, 6, 8 after the exposure, 100 cells were plated into 6-well plates (100 cells per well, 1 plate for each sample) and incubated at 37°C for 7 days to form colonies. Cells were stained with 1% methylene blue and colonies were counted manually. The viability of cells was determined at the time of each mutation harvest and calculated based on the number of colonies versus the number of inoculated cells.

Mutant frequency was calculated according to the formula:

$$ Mutant\  frequency\ \left(\times 10\hat{\mkern6mu} 6\right)=\frac{number\  of\  mutant\  colonies}{number\  of\  surviving\  inoculated\  cells} $$

Methyl methanesulfonate (MMS; 0.03 mM; 30 min) (Sigma) was used as the positive control.

### Statistical analysis

Data are expressed as mean ± SD of two-four independent experiments. Significant differences between untreated controls and treatment groups were calculated using one-way analysis of variance (ANOVA) and Tukey’s posthoc tests. To estimate IC_50_ values, a nonlinear regression analysis was used to fit a four parametric logistic curve. Graph Pad Prism software, Microsoft® Excel and Daniels XL toolbox software were used.

## Results

### Synthesis and characterization

**Figure 1:**
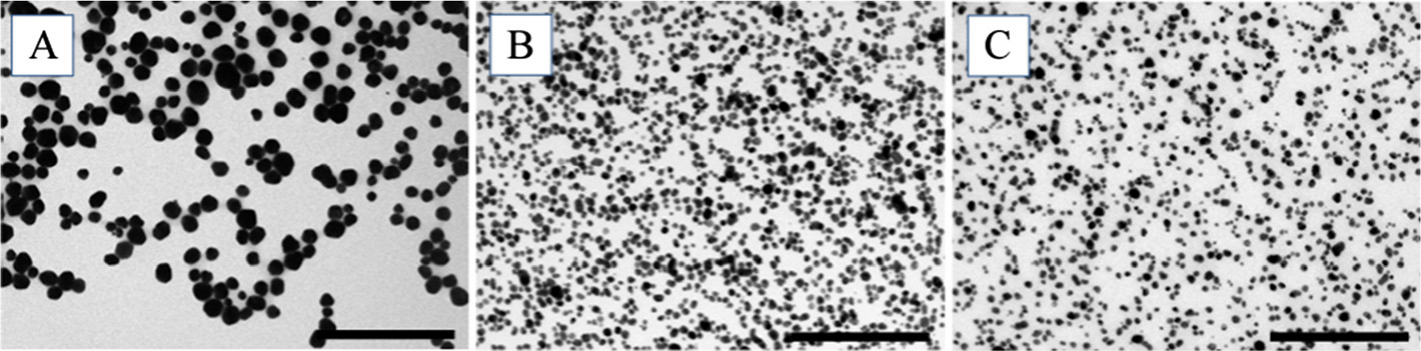
**TEM characterization of pristine Ag ENMs** **: (A)** **Ag ENMs 200 nm** **, (B)** **Ag ENMs 80 nm, (** **C)** **Ag ENMs 50 nm;** **scale bar** **: 1 μm.**

PVP-stabilized Ag ENMs were produced with the desired sizes (50, 80, 200 nm). The results of the ENM characterization in water (as synthesised) are summarized in Table 1. SEM and TEM images show a quasi-spherical shape and good monodispersity of the ENMs (Figure 1, Additional file 1: Figure S1, Additional file 2: Figure S2 and Additional file 3: Figure S3). The monodispersity was additionally proven by DLS (Table 1) and analytical centrifugation (AC) measurements (Additional files 1, 2 and 3). The XRD spectra show the crystalline nature of the Ag ENM. The presence of PVP immobilized onto the ENMs surface was indicated by XPS and SIMS analysis. XPS data also demonstrated that all surface atoms are in the Ag^0^ state, confirming the absence of Ag ions at this time point. (Additional file 1: Figure S1, Additional file 2: Figure S2 and Additional file 3: Figure S3).

**Table 1:** **Physicochemical characteristics of pristine Ag ENMs**

**Name**	**Ag ENMs 50 nm**	**Ag ENMs 80 nm**	**Ag ENMs 200 nm**
**Shape**	quasi spherical	quasi spherical	quasi spherical
**Concentration**			
**[% wt/wt]**	1	1	1
**[ENMs/ml]**	3.91×10^13^	1.03×10^13^	1.94×10^12^
**Specific surface area** ** [m** ^**2**^ **/g]**	1.37 × 10^1^	7.56	3.87
**Size/** **size distribution & aggregation/** **agglomeration state [** **nm]**	DLS: 74.5 ± 1.2	DLS: 101.3 ± 1.5	DLS: 272.5 ± 2.2
	PDI= 0.130	PDI= 0.115	PDI= 0.136
	TEM: d_50_ = 55; d_90_ = 62	TEM: d_50_ = 78; d_90_ = 96	TEM: d_50_ = 168; d_90_ = 255
	AC: d_50_ = 43; d_90_ = 78	AC: d_50_ = 77; d_90_ = 100	AC: d_50_ = 150; d_90_ = 380
**Crystal structure**	cubic	cubic	cubic
**Surface Chemistry [Atom%]**	XPS:	XPS:	XPS:
	C:48.6, Ag:25.6, O:15.9, N:7.7, Na:2.2	C:59.1, O:17.5, Ag:15.9, N 7.5	C:58.1, O:18.0, Ag:15.4, N:7.5, Na:1.0
	SIMS:	SIMS:	SIMS:
	Ag, Na, K, Ca, C_x_H_y_O_z_	Ag, Cl, C_x_H_y_O_z_	Ag, Na, K, C_x_H_y_O_z_
**Surface charge** ** [mV]**	- 17.5 ± 0.5	- 12.5 ± 0.5	- 5.5 ± 0.5
**pH**	5.9	5.9	6.0

The ENMs analyzed by DLS in biological medium supplemented with 10% FBS did not show significant changes in size distribution which confirmed that PVP prevents ENM agglomeration in protein rich media [34] and also prevents dissolution of the ENMs over the relevant timescales (48 h).

### Uptake and subcellular localisation of Ag ENMs of different sizes

**Figure 2:**
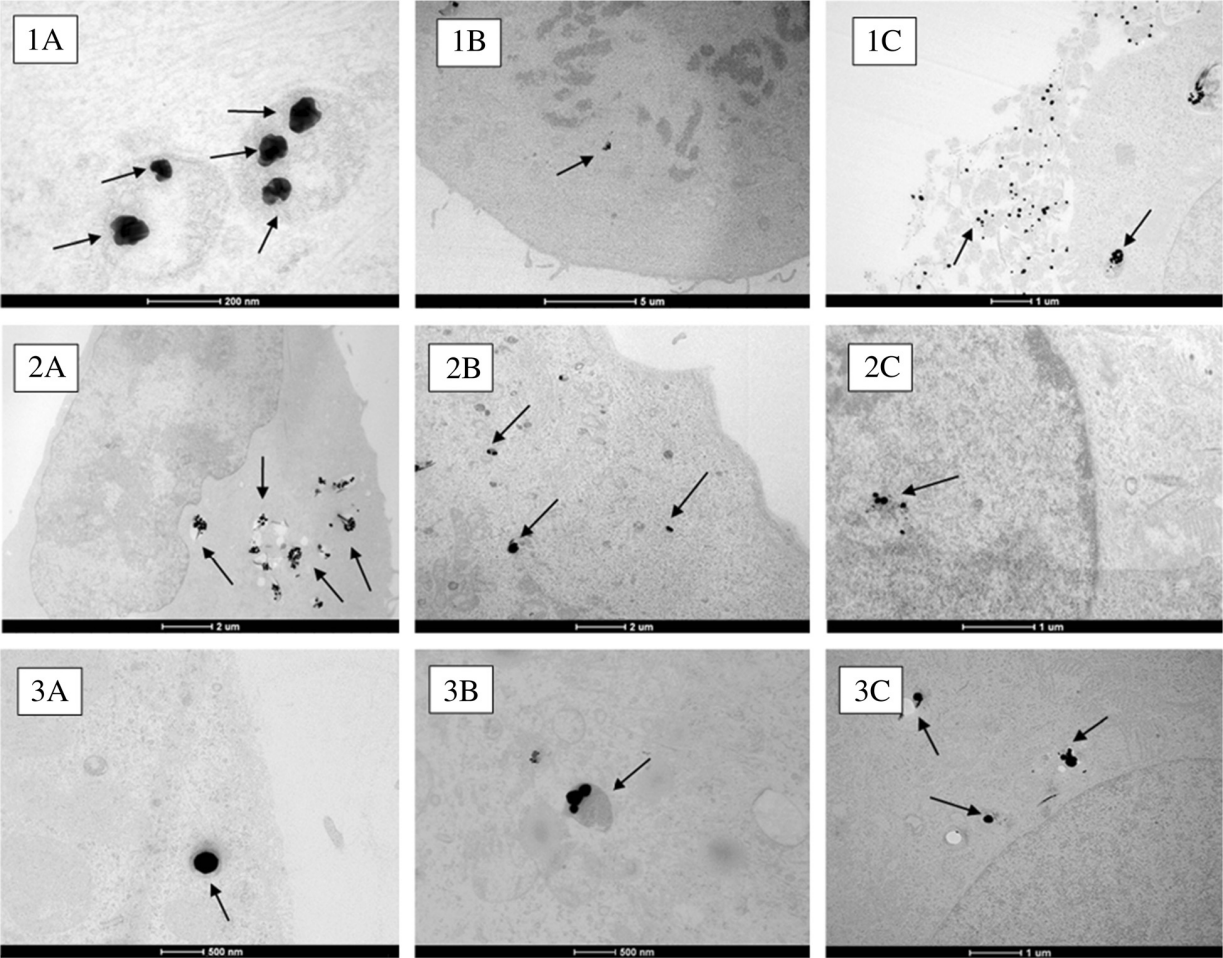
**Uptake of: (** **1)** **Ag ENMs 50 nm: (** **A)** **in cytoplasmic vesicle after 2 h exposure; (** **B)** **in close proximity to chromosomes/** **chromatin in mitotic cells after 24 h exposure** **, (C)** **outside the cell/** **associated with cellular debris (** **48 h exposure** **); (2)** **Ag ENMs 80 nm: (** **A)** **in cytoplasmic vesicle after 24 h exposure, (** **B)** **in lysosomes after 48 h exposure; (** **C)** **in nucleus after 48 h; (** **3** **) Ag ENMs 200 nm (** **A)** **in cytoplasmic vesicles after 2 h exposure; (** **B)** **in lysosomes after 24 h exposure, (** **C)** **in vesicles localised close to the nuclear membrane (** **after 24 h exposure).**

The uptake and subcellular localisation of the Ag ENMs was confirmed using TEM. Firstly, the images confirmed that ENMs of all three sizes tested were taken up by A549 cells and retained their particulate nature over the exposure duration of 48 hours (Figure 2). Ag ENMs 50 nm (Figure 2.1A) were rapidly taken up by cells, and after just 2 h of exposure the ENMs were found inside the cells in dark-coloured vesicles, presumably lysosomes. Some single ENMs were localised in vesicles, very close to the cell surface. Some of those vesicles can be interpreted as endocytotic, *clathrin*-*coated* vesicles, which indicates an active mechanism of Ag ENM uptake. After 24 and 48 h exposure, Ag ENMs 50 nm were observed to form larger clusters in lysosomes (Figure 2.1B,C). The Ag ENMs 50 nm were not observed to interact directly with mitochondria in any of the tens of images assessed, although in some cases vesicles containing Ag ENMs 50 nm were found in close proximity to those organelles. Ag ENMs 50 nm were not found inside the nucleus, although in the case of mitotic cells, where the nuclear membrane was disintegrated, they were found close to the chromosomes/chromatin. Ag ENMs 80 nm (Figure 2.2A) were also found in lysosomes after 2 h of exposure, although many ENMs (more than in the case of Ag ENMs 50 nm) were found in small, presumably endocytic vesicles. After 24 h exposure, a large number of Ag ENMs 80 nm was observed in lysosomes, where they formed big clusters. After 48 h, most ENMs were located in lysosomes, although in one case ENMs were observed within the nucleus. A smaller quantity of Ag ENMs 200 nm were observed inside the cells compared to cells treated with Ag ENMs 50 and 80 nm, consistent with literature for other ENMs but also likely related to the lower numbers of ENMs exposed at constant mass. After 2 h of exposure, Ag ENMs 200 nm were found in cytoplasmic vesicles or on the cell surface. After 24 h (Figures 2.3B,C) exposure, Ag ENMs 200 nm were found in cytoplasmic vesicles. No direct interactions with other organelles were observed. However, some vesicles containing ENMs were found close to the nuclear membrane. After 48 h, much fewer ENMs 200 nm were found inside the cells compared to the smaller sized ENMs. ENMs were still localised mainly in cytoplasmic vesicles (not shown).

### Effect of Ag ENM size on cytotoxicity and cell proliferation

**Figure 3:**
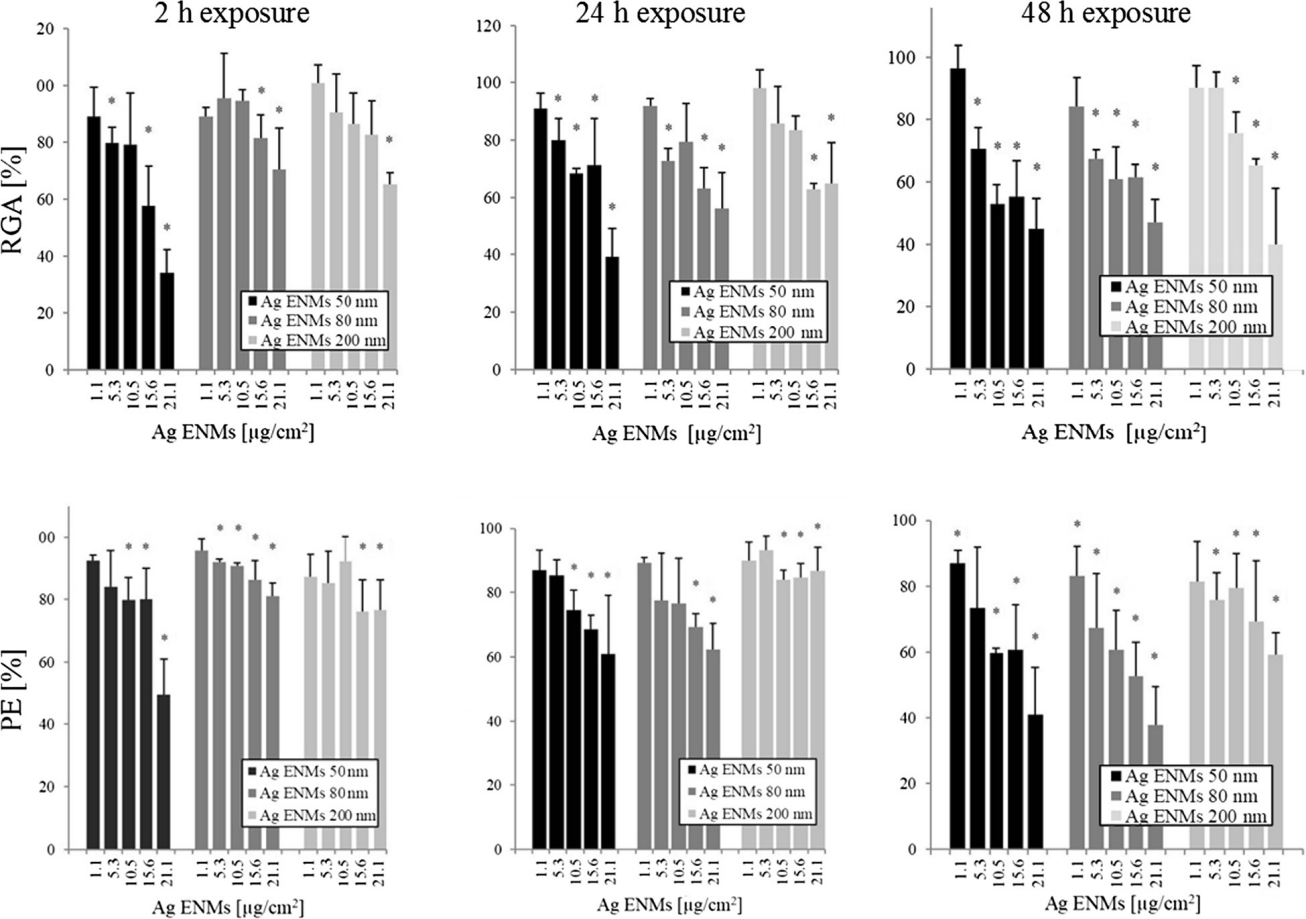
**Cytotoxic effects of 50** **, 80 and 200 nm Ag ENMs on A549 cells measured as Relative growth activity** **(RGA)** **and Plating efficiency** **(PE).** Relative growth activity (Upper figures): Cells were treated with 5 concentrations (μg/cm^2^) of Ag ENMs for 2, 24 and 48 h and cell number was counted immediately following staining. Plating efficiency (Lower figures): Cells were treated with 5 concentrations (μg/cm^2^) of Ag ENMs for 2, 24 and 48 h. Immediately after the exposure 100 cells per dish was inoculated and the number of cell clones was calculated after 10 days of incubation. Note that ENMs that had been internalised during the initial exposure remained in the cells and were diluted only by cell division over the 10 days. Columns represent cytotoxicity relative to 100% of control. The data are expressed as mean ± SD of three independent experiments. *a statistically significant (p < 0.05) difference from the unexposed (control) cells.

The effect of Ag ENMs with 3 different sizes and varying concentrations on cellular toxicity and proliferation was examined after 2, 24, 48 h exposure using PE and RGA. Data are presented with respect to the control cells that had no Ag ENM treatment (negative control). A clear concentration response was observed for all tested ENMs. On a mass basis, Ag ENMs 50 nm were considered the most cytotoxic of the tested materials (Figure 3), already evident during the 2 h exposure period in both PE and RGA tests. Conversely, expressing the data as ENMs/cm^2^ or ENMs cm^2^/cm^2^, we observed the reverse trend, where from the three tested materials, Ag ENMs 200 nm gave the highest toxic response in A549 cells (Additional file 4: Figures S5 and S6). To highlight this ambiguity in dosimetry, an IC_50_ was calculated for each system, and is summarized in Table 2. There were no statistically significant differences between IC_50_ values calculated in mass units [μg/cm^2^]. However, IC_50_ values (RGA) expressed as number of ENMs [ENMs*10^11^/cm^2^] or surface area of ENMs [ENMs cm^2^/cm^2^] were found to be several-fold less for Ag ENMs 200 nm compared with Ag ENMs 50 and 80 nm. Thus, a smaller number of the larger Ag ENMs can induce 50% impedance of cell proliferation compared with ENMs with sizes 50 and 80 nm.

**Table 2:** **IC**_**50**_ **values related to exposure to Ag ENMs for Relative growth activity** (**RGA**) **and Plating efficiency** (**PE**) **assays in A549 cells** (**Time of exposure 2**, **24**, **48 h**)

		**Ag ENMs 50 nm**	**Ag ENMs 80 nm**	**Ag ENM 200 nm**
**Relative growth activity**				
[μg/cm^2^]	T = 2 h	16.8 ± 1.71	24.42 ± 1.82	23.68 ± 1.51
	T = 24 h	23.68 ± 11.42	37.53 ± 17.58	36.37 ± 12.84
	T = 48 h	15.94 ± 1.33	26.09 ± 11.63	21.71 ± 7.41
[ENMs × 10^11^/cm^2^]	T = 2 h	6.57 ± 0.6	2.51 ± 0.19	0.46 ± 0.03
	T = 24 h	9.26 ± 4.47	3.86 ± 1.81	0.75 ± 0.25
	T = 48 h	6.23 ± 0.52	2.69 ± 1.2	0.42 ± 0.14
[ENMs cm^2^/cm^2^]	T = 2 h	2.3 ± 0.23	1.85 ± 0.13	0.61 ± 0.53
	T = 24 h	3.24 ± 1.33	2.84 ± 1.33	1.41 ± 0.5
	T = 48 h	2.18 ± 0.18	1.97 ± 0.88	0.84 ± 0.29
**Plating efficiency**				
[μg/cm^2^]	T = 2 h	35.58 ± 13.7	-	-
	T = 24 h	31.65 ± 15.81	31.65 ± 15.81	-
	T = 48 h	17.83 ± 4.5	14.25 ± 1.94	-
[ENMs × 10^11^/cm^2^]	T = 2 h	13.91 ± 5.35	-	-
	T = 24 h	12.37 ± 6.18	3.66 ± 0.41	-
	T = 48 h	6.97 ± 1.77	1.48 ± 0.2	-
[ENMs cm^2^/cm^2^]	T = 2 h	4.87 ± 1.89	-	-
	T = 24 h	4.37 ± 2.17	2.69 ± 0.3	-
	T = 48 h	2.44 ± 0.62	1.08 ± 0.15	-

-IC_50_ could not be estimated. IC_50_ values were calculated for 3 different concentration characterization units: number of ENMs (ENMs × 10^11^/cm^2^), surface area of ENMs (ENMs cm^2^/cm^2^) and mass of ENMs (μg/cm^2^) per exposure surface.

### Observation of cellular morphology

Morphological changes of A549 cells exposed to 50, 80, 200 nm Ag ENMs were observed using an inverted optical microscope (Additional file 4: Figure S4). After 24 h incubation with all three sizes of Ag ENMs, no changes of cellular morphology were observed, compared to the untreated cells (negative control). Detached or rounded cells were not observed. However, Ag ENMs attached to the cells can be observed as dark spots on the cell surface.

### Release of inflammation-related proteins

**Figure 4:**
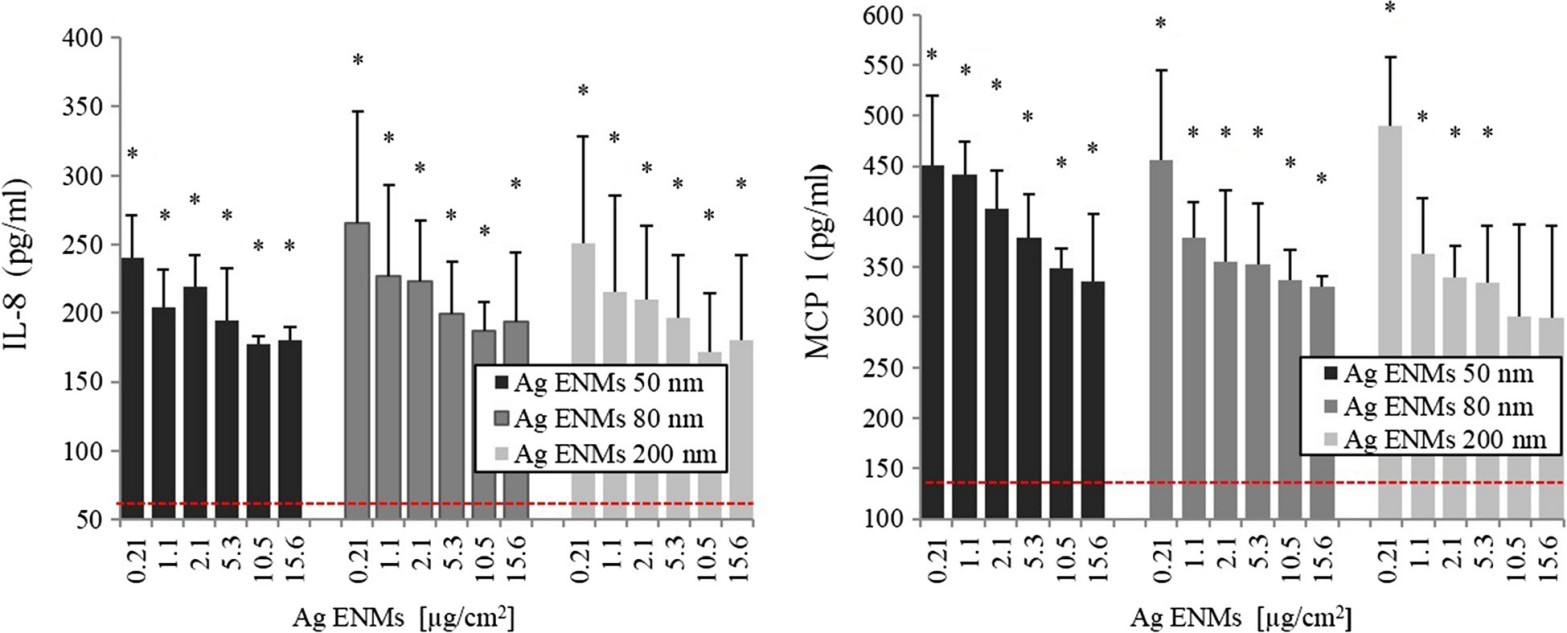
**Induction of IL** **-8 and MCP-** **1 in A549 cells exposed to Ag ENMs 50,** **80 and 200 nm.** Cells were treated with 6 concentrations (μg/cm^2^) of Ag ENMs for 24 h. The data are expressed as mean ± SD of at least three independent experiments. *statistically significant (p < 0.05) difference from the unexposed control. Horizontal line represents expressed level of IL8 (81.8 ± 31.5 pg/ml) and MCP-1 (143.3 ± 26.68 pg/ml) in untreated cells. TNFα (20 ng/ml) as a positive control (IL-8) gave 386.5 ± 87.92 pg/ml and (MCP-1) gave 528.3 ± 134.52 pg/ml.

The effect of 50, 80 and 200 nm Ag ENMs in the concentration range from 0.21-15.6 μg/cm^2^ on the cellular production of inflammatory cytokines (IL-8 and MCP-1) by A549 cells was measured using the ELISA assay (Figure 4). Reverse concentration response trends were observed for all tested materials. However, statistically significant differences were not found between any of the tested Ag ENMs when plotted in mass units.

### Detection of DNA strand breaks and oxidised lesions

**Figure 5:**
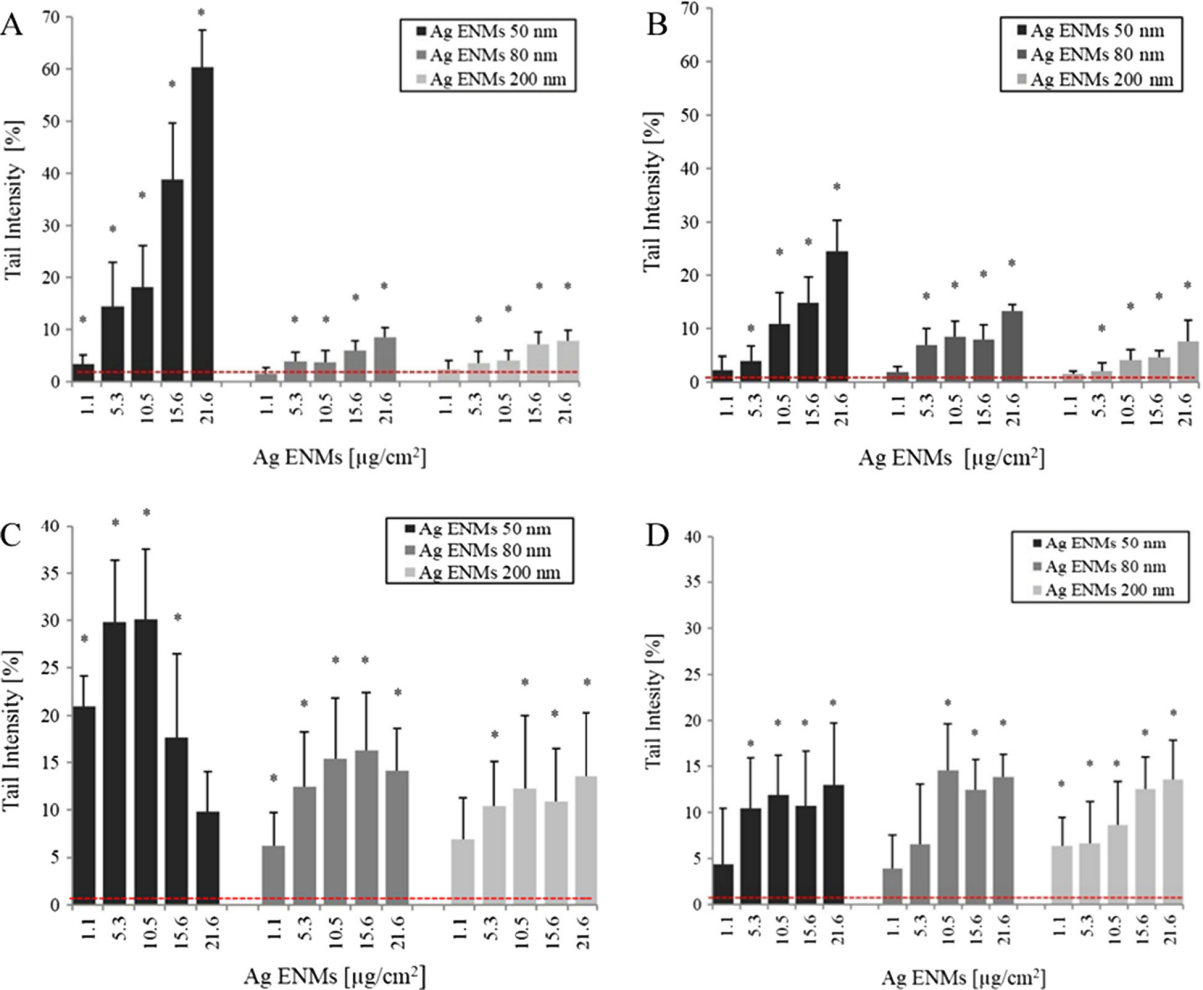
**Level of DNA damage** **– strand breaks** **(A,** **B)** **and oxidised DNA lesions expressed as NET FPG (** **C,** **D)** **in A549 cells exposed to different concentrations of Ag ENMs (** **μg/** **cm** ^**2**^ **) with sizes 50,** **80,** **200 nm for 2 h (** **A,** **C)** **and 24 h** **(B,** **D).** NET FPG was estimated as FPG-sensitive sites minus strand breaks, representing altered purines. Horizontal lines represent level of strands breaks (SBs)/oxidised bases (NET FPG) in untreated cells (**A**: 1.36 ± 0.84; **B**: 0.76 ± 0.5; **C**: 1.99 ± 1.7; **D**: 3.3 ± 1.56% tail DNA). The data are expressed as mean ± SD of three independent experiments. *significant (p < 0.05) difference from the unexposed control. Hydrogen peroxide (50 μM, 5 min in PBS), a positive control (SBs) gave 38.72 ± 6.1% tail DNA. Photosensitiser Ro19-8022 (1 μM in PBS, 5 min, on ice), a positive control for oxidised DNA lesions (NET FPG) gave 33.2 ± 10.96% tail DNA.

The standard alkaline Comet assay was employed for detection of single and double strand breaks, and a modified version with FPG was used to detect oxidized DNA lesions (Figure 5). A significant concentration response was observed at all time points and for all ENMs tested. The strongest effect was observed in cells treated with 50 nm Ag ENMs at all tested concentrations, and our data demonstrated that the genotoxic potential of Ag ENMs is both concentration and size dependent. No statistically significant difference was found between the level of DNA damage induced by Ag ENMs 80 and 200 nm at any exposure time (Figure 5A and B or Figure 5C and D). An increased level of oxidised DNA lesions was also observed in all treated groups (Figure 5C and D), but again the strongest effect was demonstrated in cells exposed to the smallest ENMs at the shortest exposure time. A significant level of DNA oxidation was already seen in cells exposed at the lowest concentration (1.1 μg/cm^2^) of Ag ENM 50 nm. The high genotoxic response to ENMs 50 nm can be coupled with the higher number of ENMs compared to the number of 80 nm and 200 nm ENMs. Re-calculating the data from mass units [μg/cm^2^] to number of ENMs [ENMs*10^11^/cm^2^] or surface area of ENMs [ENMs cm^2^/cm^2^] showed that Ag 50 nm ENMs are the most genotoxic only at short time exposures – 2 h, but for longer treatment times the highest DNA damaging effect was observed with 200 nm Ag ENMs. Supplementary figure (Additional file 4: Figure S7 and S8).

### Impact of Ag ENMs on induction of gene mutations

**Figure 6:**
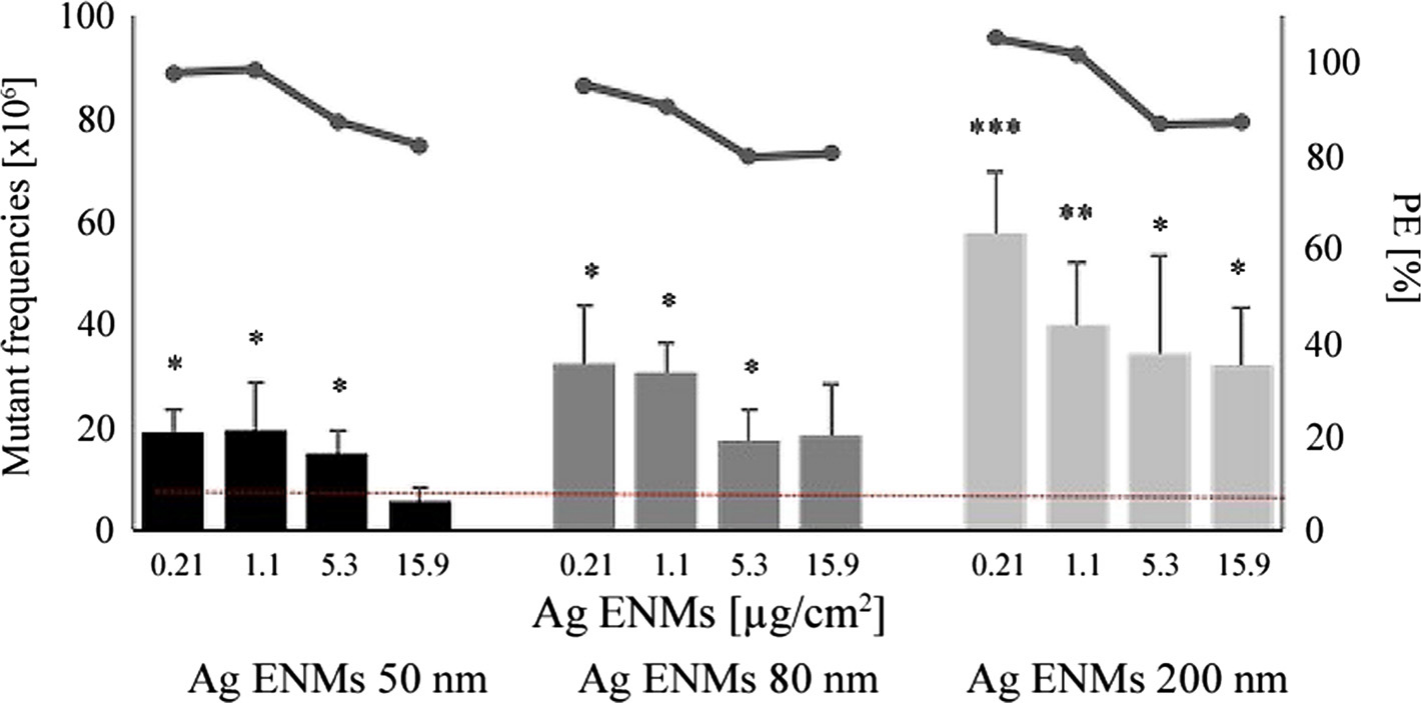
**Effect of 24 h treatment with 50,** **80 and 200 nm Ag ENMs** **(0.21** **-15.6 μg/** **cm** ^**2**^ **) on induction of** ***hprt*** **gene mutations in V79-** **4 cells (** **bar graphs,** **left hand scale).** The mutant frequencies (x10^6^) are expressed as the mean ± SD of two independent experiments, with two independent harvests per experiment. Horizontal line shows *hprt* gene mutant frequency in untreated cells (8.47 ± 3.83). MMS (0.03 μM, 30 min), a positive control gave 80.03 ± 22.13 *hprt* gene mutations. Significant difference from unexposed control (*p < 0.05, **p < 0.01, ***p < 0,001). Cytotoxic effects – Plating efficiency (PE) (horizontal marker line, right hand scale) is expressed as the mean of two independent experiments. Results represent cytotoxicity relative to 100% of the negative control. Cytotoxicity of MMS has not been observed (PE = 97.99 ± 6.14%).

The mutagenic potential of 50, 80, 200 nm Ag ENMs was examined using the *hprt* gene mutation assay according to OECD guideline 476. Two independent experiments, each with two mutation harvests, were performed (Figure 6A). Ag ENM 200 nm were observed to be the most mutagenic (p < 0.001), at all concentrations tested. The data are presented in mass units; however, this pattern holds true when plotted as number of ENMs, or by surface area. We observed the highest frequency of *hprt* mutants in the first experiment; mutant frequency was 9.6 ± 5.25 times higher than the negative control. The frequency of induced mutants in several groups was comparable with the mutant frequency found with the positive control (MMS). For cells treated with Ag ENMs 50 and 80 nm there was a trend towards higher mutant frequency of 80 nm ENMs compared with 50 nm; however, this was not significant.

## Discussion

The increasing number of consumer products containing Ag ENMs stimulates researchers to investigate their potential adverse health effects. Risk assessment strategies for ENMs require relevant *in vitro* toxicology research of new materials, with special attention given to the mechanisms of toxicity and the use of representative reference materials to correlate the toxic effect of ENMs with their specific physico-chemical properties. Investigations so far have shown that the toxic potential of ENMs is related to their physico-chemical properties, such as shape, size, charge, aggregation stage and chemical composition [35-40].

Several researchers reported that cytostatic, cytotoxic and genotoxic effects of Ag ENMs are mostly mediated via oxidative stress, e.g. via high production of reactive oxygen species (ROS) [41]. The induced oxidative stress may affect cell cycle, resulting in G2/M phase arrest which leads to apoptosis and causes cell death [12]. The observed toxicity of Ag ENMs is thus the result of ENMs uptake and localised release of high concentrations of Ag ions inside the cells, known as the Trojan horse mechanism [42].

PE and RGA were selected to study proliferation and direct cytotoxic effects of Ag ENMs as well as cellular viability. The advantage of these tests in nanotoxicology is that there is no or little risk of interference between the assay and the ENMs [23,43]. We demonstrated that cytotoxic and cytostatic effects were observed after exposure to all ENMs tested, and that this response increased with the duration of the exposure and the ENM concentration. The strong positive effects from Ag ENMs 50 nm observed after the short 2 h exposure in the RGA assay is mostly related either to cell membrane damage (which can be repaired) or necrosis (cell dying) and thus it reflects several toxic effects. For the PE assay, any ENM taken up by the cells during the exposure remain in the cells, although gradual dilution of the ENM load occurs as a result of cell division as the ENMs are split between daughter cells [44]. Thus, differences in the IC_50_ values between the PE and RGA assays suggest that most of the initial (early time) damage can be repaired after the end of exposure, when the cells are further cultivated in ENM free medium and the initial ENM concentration is diluted over time due to cell division. Nevertheless, reduction of viable populations to lower than 60% was found at higher Ag ENM concentrations. No significant differences were found between 80 and 200 nm Ag ENMs, albeit the ENMs numbers were lower here, and the trends observed also mirrored those of the 50 nm Ag ENMs.

Genotoxic effects of Ag ENMs have been extensively studied, and many researchers reported that Ag ENMs can induce different types of DNA damage, including strand breaks, DNA oxidation, bulky DNA adducts, formation of micronuclei and chromosome aberrations [14,26,45]. Genotoxicity testing in a regulatory perspective requires a battery of tests addressing these different genotoxic and mutagenic endpoints. The Comet assay is one of the most commonly used methods to study specific DNA lesions such as single and double strand breaks, oxidation, alkylation of DNA, cross links, and can be successfully applied in nanogenotoxicology [23,46,47]. According to recent literature, the toxic effect of ENMs may be coupled with ROS production. For this reason, we used a modified protocol including the incubation of nuclei embedded in gels with FPG to detect oxidised purines, predominantly 8-oxo-guanine, one of the most pre-mutagenic lesions [48].

Previous studies reported a possible genotoxic potential of Ag ENMs, showing that Ag ENMs can induce DNA breaks and DNA oxidation. However, differences between nano- and microsized Ag ENMs were never taken fully into consideration.

Our results report significant increase of DNA damage after 2 h exposure to Ag ENMs 50 nm. However, the two highest concentrations tested also substantially reduced the viable cell populations, which means that the higher level of DNA damage might be a result of potential apoptosis or necrosis rather than genotoxicity. Due to the high cytotoxicity and almost saturated level of strand breaks, a significant increase of oxidised purines was not found at these ENM concentrations. Slight but significant increases in DNA breaks were found in both 80 and 200 nm Ag ENMs treated cells at both short (2 h) and long (24 h) exposure times, with no differences observed between the two ENM sizes. An increased level of DNA breaks was found mostly at the cytotoxic concentrations (≥5.3 μg/cm^2^).

On the other hand, an increased number of oxidised DNA lesions was observed in viable cell populations, which correlates with the fact that Ag ENMs have an impact on ROS production in cells. These results also demonstrate the importance of studying specific DNA lesions such as oxidised purines (the majority of these lesions are 8-oxo-guanins) that could be detected by FPG, or OGG1 glycosylases [48]. A decreasing level of DNA damage was observed between the 2 and 24 h exposures for cells treated with 50 nm Ag ENMs. This could be due to removal of some parts of damaged cells during the washing step after exposure, be the result of the ENMs distribution between new generations of cells, or be a result of DNA repair and cellular recovery. In our previous nanotoxicology studies we investigated the potential for oxidized DNA lesions caused by Ag ENMs to be reduced by the presence of antioxidants. We used plant extract from *Gentiana asclepiadea*, rich in substances with high antioxidant abilities such as a swertiamarin, mangiferin and homoorientin [14,49]. DNA oxidation induced by Ag ENMs was efficiently diminished by these plants extracts. Additionally, we demonstrated that the presence of antioxidants significantly enhanced DNA repair. We measured levels of DNA lesions at different time points after exposing cells to Ag ENMs. Cells incubated with plant extract from *Gentiana asclepiadea* had significantly lower level of DNA damage compared to the control group, which proved that the presence of antioxidants can inhibit the harmful effects of Ag ENMs [49]. To investigate the mutagenic potential of Ag ENMs we applied the *hprt* gene mutation assay. In genotoxicity testing the most used assay for mutagenicity is the bacterial Ames test. However, this assay has serious limitations for ENM mutagenicity testing because of the size of bacteria (not much bigger than some ENMs), and due to the presence of the cell wall which results in limited or no uptake of ENMs as they cannot pass through the bacterial wall [30]. Tedser *et al*. did not find any mutagenic effect of PVP stabilized Ag ENMs with different sizes and shapes in Ames/Salmonella typhimurium assay [50]. Another two studies using the Ames test also reported no mutagenic effects of Ag ENMs [51,52] although these most likely show false negative results. The *hprt* gene mutation assay has clear advantages for testing of ENMs as it is based on a mammalian cell model that is closer to human physiology. To date, there are only a few publications on mammalian mutagenicity of ENMs published. Kim *et al*. applied the thymidine kinase (*tk*^+/−^) gene mutation assay based on the mammalian L5178Y cell line, and reported no/slight but not significant increase of thymidine kinase mutants in cells treated by Ag ENMs [53]. However, it is difficult to interpret this study because of differences in the materials used, and the lack of detailed ENM characterization.

In the study reported herein, a high number of *hprt* mutants were observed, in viable cell populations, with all tested Ag ENMs. Interestingly, the number of mutants decreased with increasing ENM concentration. This might be due to larger genetic malformations being induced by higher ENM concentrations that consequently reduce cell viability of the mutant cells with the result that lower mutant frequency is observed. Wang *et al*. reported a 2.5 fold increase in the mutant frequency in WIL2-NS cells treated by ultrafine TiO_2_ ENMs, but the positive effect was only observed at the higher tested concentration [54]. Also diesel exhaust NMs and carbon black ENMs were shown to be mutagenic, significantly increasing the mutant frequency in FE-1Muta™ mouse lung epithelial cells [55,56]. A similar reverse concentration response has been observed in a separate study performed in our lab using gold ENMs, and again is suggested to result from more extreme mutations occurring at higher concentrations resulting in lower mutant cell viability and thus lower observation of mutant frequency [Porredon C, El Yamani N, Lapuente J, López DR, Coloma A, Borràs M, Dusinska M: In vitro genotoxicity study of coated and uncoated gold nanoparticles evaluated by the Mammalian cell *hprt* gene mutation assay in Chinese hamster V79 cells. Manuscript in preparation].

Correlation of results from the Comet assay and the *hprt* gene mutations assay is challenging as these two tests measure different endpoints and require different cell types, cell seeding densities and exposure durations, leading to different ENM exposure regimes. In the Comet assay we measured several types of DNA damage, DNA strand breaks and oxidized DNA lesions that are considered as pre-mutagenic lesions. The Comet assay thus detects transient DNA lesions that can be repaired, or can result in mutations. Also, the outcomes might be affected by several other biological processes such as apoptosis or necrosis. With the Comet assay it is crucially important to measure DNA damage at non-cytotoxic or slightly cytotoxic ENM concentrations to ensure that the effect observed is not related to cytotoxicity. Compared to the Comet assay, the *hprt* gene mutation test detects permanent changes in the nucleotide sequence of the genetic material inherited or raised by incorrect nucleotide base pairing during replication or by erroneous repair. We performed the *hprt* mutation test following OECD guideline which recommend V79-4 cells (as A549 cells are not a suitable cellular model for this test). V79-4 cells have a shorter cell cycle (12 h) compared to A549 (24 h), and thus due to faster cell division, the total number of ENMs is diluted in V79-4 cells between new, daughter cells more rapidly which results in different concentrations during exposure compared to A549 cells.

ENMs can induce mutagenicity via direct or indirect mechanisms. Indirect mechanisms involve ROS production or inflammation [57]. Hackenber and Greulich demonstrated that Ag ENMs can stimulate release of inflammatory markers (IL-8 and IL-6) but only at non cytotoxic ENM concentrations. Additionally, they found that at cytotoxic and genotoxic concentrations of Ag ENMs, production of cytokines is inhibited [18,58]. This same phenomenon was observed at the size depended toxicity study of PVP stabilized Ag ENMs with nominal sizes 4, 20 and 70 nm [59]. Production of inflammatory markers in our study also decreased with increased concentrations of Ag ENMs, as already reported by Kermanizadeh *et al*. and by Mahl *et al*. using a panel of different ENMs [60,61]. However, we did not find significant differences between all three tested Ag ENMs, nor very significant concentration-responses, suggesting that this is not a sensitive marker for Ag ENMs. Although we did not study possible interference of PVP coated ENMs with ELISA, we found that PVP immobilized at ENM surfaces reduces protein binding, suggesting that the likelihood of such interference is low [33].

The significant differences between nano and non-nano sized Ag in both cytotoxicity and genotoxicity can be explained by the intercellular localisation and number of ENMs taken up by cells. Smaller ENMs were found close to chromatin (Ag ENMs 50 nm) or mitochondria and nucleus (Ag ENMs 80 nm). Smaller ENMs are also taken up faster and to a higher extent compared to micro sized (Ag ENM 200 nm) materials. The lower uptake of 200 nm Ag ENMs compared to 50 nm ones is consistent with the lower particle numbers at constant mass and the lower efficiency of endocytotic receptors for larger entities. Recently, Varela *et al*. studied size-dependent uptake rates of fluorescently labelled carboxylated polystyrene ENMs (20, 40 and 100 nm) in two different cell types (A549 and 1321 N1 human astrocytoma), keeping the number of ENMs per unit volume constant for all sizes [62]. They showed that 40 nm carboxylated polystyrene ENMs were internalized faster than 20 nm or 100 nm ENMs in both cell lines studied, suggesting that there is a privileged size gap in which the internalization of ENMs is higher. Interestingly, previous TEM studies, using both polystyrene and silica ENMs have not shown any evidence of internalised ENMs existing outside of endosomal, lysososal or multi-lamellar structures nor was there any evidence of cellular damage in response to the presence of the ENMs such as that observed with the Ag ENMs 50 nm in Figure 2.1C [63,44].

Previous studies investigated different mechanisms involved in Ag ENMs toxicity and showed that toxicity of Ag ENMs is size dependent and such size dependence can be also correlated with release of ions [27]. Smaller Ag ENMs released more ions than bigger ENMs. However, the ion fraction released during exposure did not have an impact on cell toxicity [27]. Among our extensive ENM characterization, XPS data demonstrated that all of the surface atoms were in the Ag^0^ state, confirming the absence of Ag ions in the Ag ENMs solution in contrast with many published studies [10,11,14,15,18,51,58]. In an additional study (published separately) we investigated the kinetics of Ag ENMs dissolution during storage, and found no significant changes in Ag ENMs physico-chemical properties, including dissolution or aggregation/agglomeration over 6 months in Ag ENMs with neutral charge [Izak-Nau E, Huk A, Reidy B, Uggerud H, Vadset M, Eiden S, Voetz M, Duschl A, Dušinska M, Lynch I: Impact of Storage Conditions and Storage Time on Silver Nanoparticle Physicochemcial Proper ties and Implications for Biological Effects. Manuscript in preparation]. This finding was also confirmed by Kittler *et al*. [32]. However, Ag ENMs can release Ag ions during exposure in cell culture medium, although literature suggests that most of the Ag ions are bound with chlorine ions present in medium and precipitate as AgCl salt [64]. Several studies show that the level of free ion fractions is between 6–20% of the applied concentration of Ag ENMs [27,33,40,65,66] and this concentration is too low to cause toxic effect. Additionally, experiments with the ultracentrifuged ion fraction obtained from PVP stabilized Ag ENMs (similar to our ENMs) have shown no effect on cell viability or genotoxicity, suggested limited dissolution of the PVP-capped Ag ENM over the relevant timeframe [24,27,40].

Comparison between the observed toxicity of nano and micro scale of Ag ENMs was studied by Park *et al*., who reported strong differences between 20 nm and 113 nm Ag ENMs in both cytotoxicity and immune regulation assays [67]. Our study shows that Ag ENMs 50 nm are more cytotoxic and genotoxic than the larger materials tested, mostly because of the higher surface area and higher number of ENMs at constant mass relative to the 80 and 200 nm Ag ENMs. However, of the Ag ENMs studied, the 200 nm Ag ENMs which had the smallest surface area and the lowest number of ENMs per volume were observed to have the strongest impact on induction of mutations (Additional file 4: Figures S11 and S12). The results from the *hprt* gene mutation assay suggest that the mode of action of genotoxicity of Ag ENMs can go via several simultaneous mechanisms, with the final impact being dependent on ENM size, concentration, type of damage induced and whether the damage can be repaired.

## Conclusion

The main goal of this study was to assess whether there is a correlation between the size of Ag ENMs and their toxic potential. We were interested to see if ENMs with the same shape, charge and chemical composition (including the stabilizing coating) but with different sizes resulted in similar toxicity responses in mammalian cells. Our results show that it cannot be generalized that Ag in nano form is always more toxic than its micro form with the same chemical composition and all other factors being equal, as suggested by Karlsson *et al*. [68]. In our study, we observed strong cytotoxic and genotoxic effects of Ag ENMs 50 nm, but from the tested materials Ag ENMs 200 nm had the most mutagenic potential. Our study demonstrates that Ag ENMs can induce DNA damage via more than one mechanism; directly by contact with chromatin or DNA [12], as also shown in TEM (Figure 2.1B and 2.2C), or indirectly by ROS production which was indicated by DNA oxidation. Our results also show that expression of ENM concentrations as number of ENMs or in terms of surface area is more representative for evaluation of toxicity of different sized ENMs than using the usual mass units.

## Additional files

Additional file 1: Figure S1. Detailed characterization of Ag ENMs 50 nm. Additional file 2: Figure S2. Detailed characterization of Ag ENMs 80 nm. Additional file 3: Figure S3. Detailed characterization of Ag ENMs 200 nm. Additional file 4: Figure S4. Light microscopy (100x) of A549 cells incubated with 50, 80, 200 nm Ag ENMs (concentration 21.2 μg/cm^2^) for 24 h. A: Negative control, B: Cells treated with Ag ENMs 50 nm; C: cells treated with Ag ENMs 80 nm; D: cells treated with Ag ENMs 200 nm. **Figure S5.** Cytotoxic effects of 50, 80 and 200 nm Ag ENMs on A549 cells measured as Relative growth activity (RGA) and Plating efficiency (PE). Concentrations of Ag ENMs expressed as ENMs cm^2^/cm^2^. **Figure S6.** Cytotoxic effects of 50, 80 and 200 nm Ag ENMs on A549 cells measured as Relative growth activity (RGA) and Plating efficiency (PE). Concentrations of Ag ENMs expressed as ENMs/cm^2^. **Figure S7.** Level of DNA damage – strand breaks and oxidised DNA lesions expressed as NET FPG in A549 cells exposed to different concentrations of Ag ENMs. Concentrations of Ag ENMs expressed as ENMs cm^2^/cm^2^. **Figure S8.** Level of DNA damage – strand breaks and oxidised DNA lesions expressed as NET FPG in A549 cells exposed to different concentrations of Ag ENMs. Concentrations of Ag ENMs expressed as ENMs/cm^2^. **Figure S9.** Induction of IL-8 and MCP-1 in A549 cells exposed to Ag ENMs 50, 80 and 200 nm. Concentrations of Ag ENMs expressed as ENMs cm^2^/cm^2^. **Figure S10.** Induction of IL-8 and MCP-1 in A549 cells exposed to Ag ENMs 50, 80 and 200 nm. Concentrations of Ag ENMs expressed as ENMs/cm^2^. **Figure S11.** Effect of 24 h treatment with 50, 80 and 200 nm Ag ENMs on induction of *hprt* gene mutations in V79-4 cells. Concentrations of Ag ENMs expressed as ENMs cm^2^/cm^2^. **Figures S12.** Effect of 24 h treatment with 50, 80 and 200 nm Ag ENMs on induction of *hprt* gene mutations in V79-4 cells. Concentrations of Ag ENMs expressed as ENMs/cm^2^.

## Abbreviations

A549: Human lung carcinoma epithelial cells
AC: Analytical centrifugation
BET: Brauner-Emmett-Teller
DLS: Dynamic Light Scattering
ELISA: Enzyme linked immunosorbent assay
ENMs: Engineered nanomaterials
FPG: Formamidopyrymidine DNA glycosylase
IC_50_: Half maximal inhibitory concentration
OECD: Organization for Economic Co-operation and Development
PE: Plaiting efficiency
PVP: Polyvinylpyrrolidone
RGA: Relative Growth Activity
SEM: Scanning electron microscopy
SBs: Strand breaks
TEM: Transmission electron microscopy
ToR SIMS: Time-of-flight secondary ion mass spectrometry IV
V79-4: Chinese hamster lung fibroblast cells
XPS: X-ray photoelectron spectroscopy
XRD: X-ray diffraction
