# Supplementary material for: Is the toxic potential of nanosilver dependent on its size?
Source: Part Fibre Toxicol. 2014 Dec 3;11:65. doi: 10.1186/s12989-014-0065-1 (PMC4274708; doi:10.1186/s12989-014-0065-1)
Supplement: Additional file 1: Figure S1. — Detailed characterization of Ag ENMs 50 nm. [file 12989_2014_65_MOESM1_ESM.pdf]

NanoTOES
Nanotechnology: Training of Experts in Safety

Material	Ag (50 nm)
**NanoTOES number**	NanoTOES_Ag_50
**Provider**	Bayer Technology Services GmbH
**Concentration**	1 % (wt/wt) in water<br>$3.91*10^{13}$ NPs/ml
**Specific Surface Area**	$1.37*10^{1}$ m^2/g
**Size/<br>Size distribution &<br>Agglomeration/<br>Aggregation state**	DLS: (Correlation Func., Number Distribution)<br>74.5 nm +/-1.2 ; PDI= 0.130<br>TEM: d50= 55 nm, d90= 62 nm<br>AC: d50= 43 nm, d90= 78 nm
**Shape**	quasi spherical
**Crystal Structure**	cubic
**Surface Chemistry**	XPS: Atom % C 48.6, Ag 25.6, O 15.9, N 7.7, Na 2.2<br>SIMS: Ag, Na, K, Ca, $C_xH_yO_z$
**pH (in water)**	5.9
**Surface Charge**	Zeta potential: - 17.5 mV+/- 0.5<br>IEP: ~ 1.3 pH

**Scanning (Raster) Electron Microscopy (SEM)**	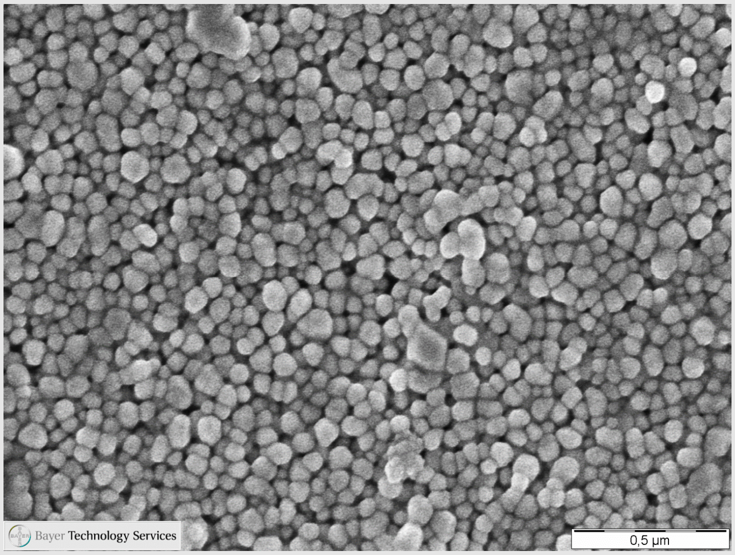
**Transmission Electron Microscopy (TEM)**	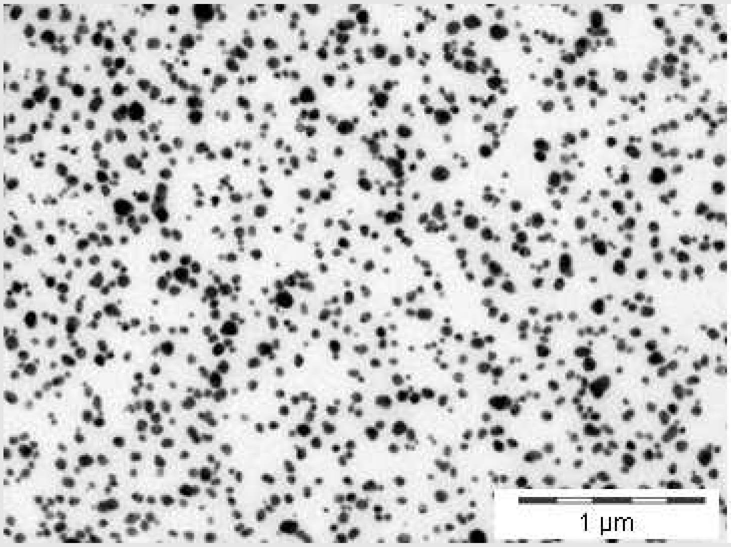 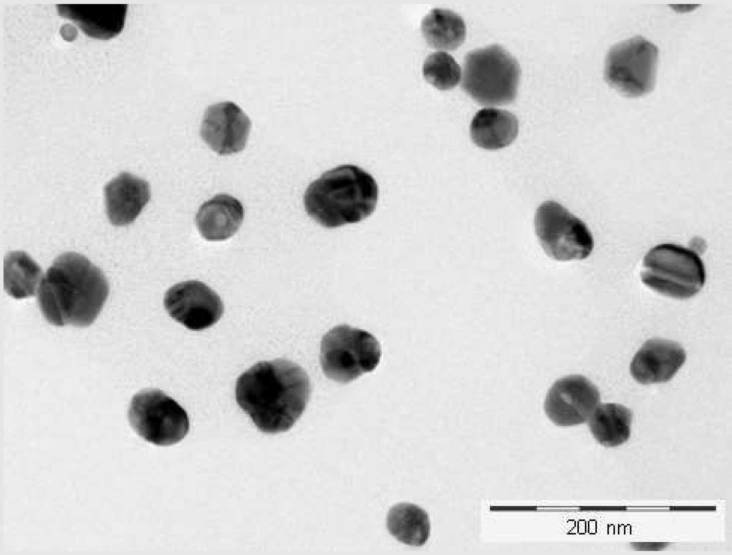

<table>
<tr>
<td>X-ray Diffraction (XRD)</td>
<td>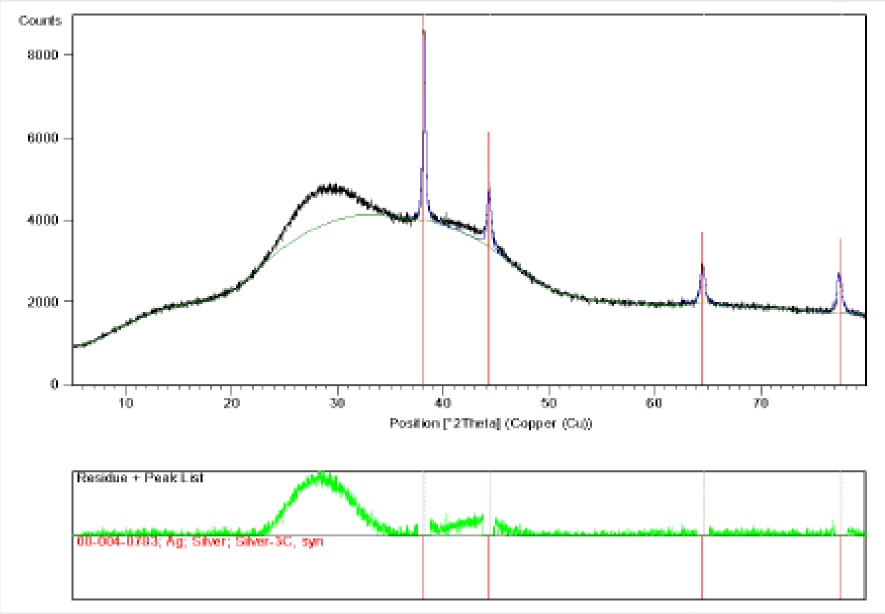
</td>
</tr>
<tr>
<td>X-ray Photoelectron Spectroscopy (XPS)</td>
<td>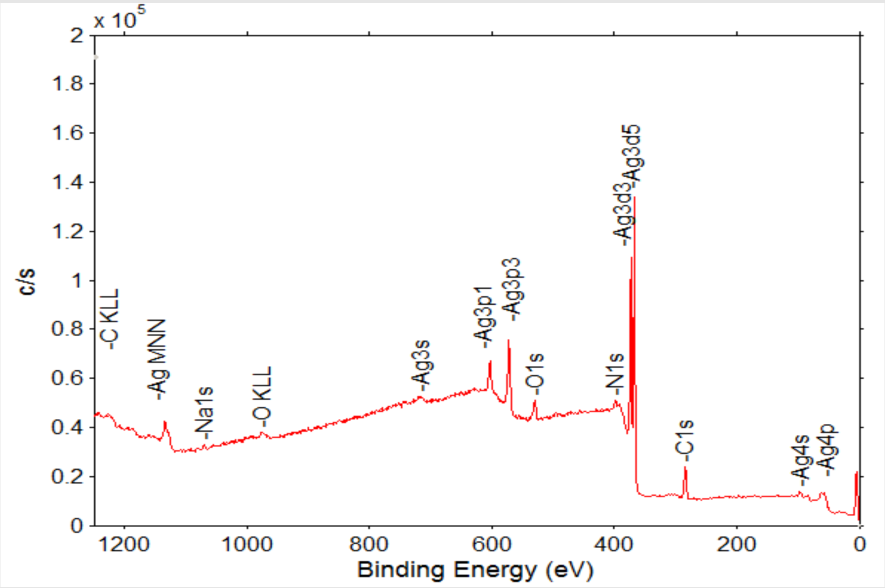
</td>
</tr>
</table>

**Analytical Ultracentrifugation (AC)**	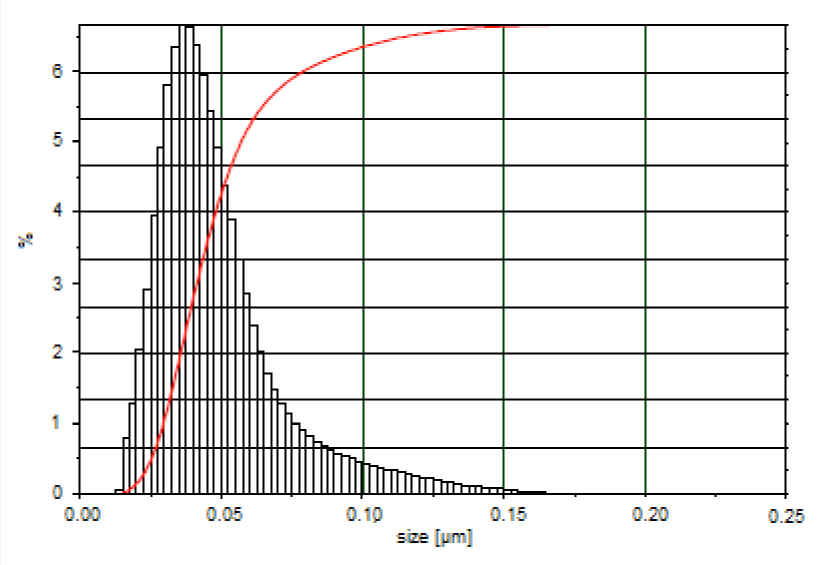
**Zeta Potential & Isoelectric Point**	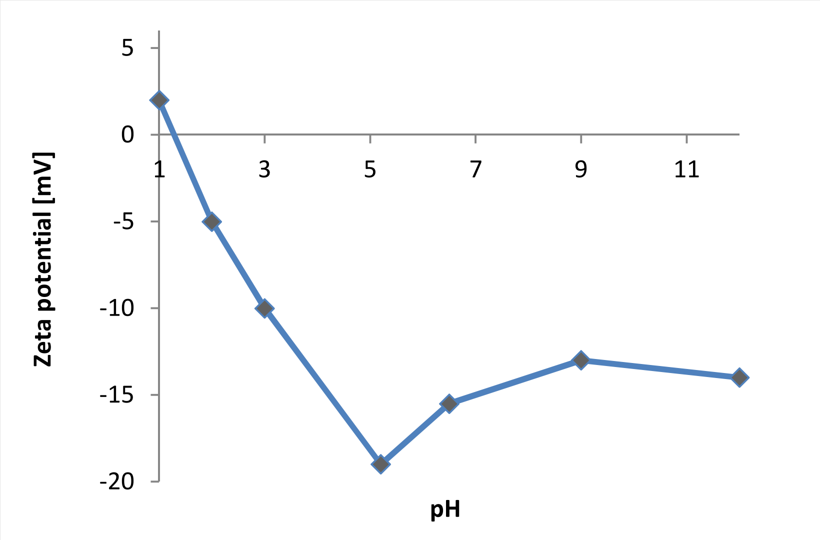
