# Supplementary material for: Is the toxic potential of nanosilver dependent on its size?
Source: Part Fibre Toxicol. 2014 Dec 3;11:65. doi: 10.1186/s12989-014-0065-1 (PMC4274708; doi:10.1186/s12989-014-0065-1)
Supplement: Additional file 2: Figure S2. — Detailed characterization of Ag ENMs 80 nm. [file 12989_2014_65_MOESM2_ESM.pdf]

NanoTOES
Nanotechnology: Training of Experts in Safety

Material Ag (80 nm)

**NanoTOES number**	NanoTOES_Ag_80
**Provider**	Bayer Technology Services GmbH
**Concentration**	1 % (wt/wt) in water<br>1.03*10^{13} NPs/ml
**Specific Surface Area**	7.56 m^2/g
**Size/<br>Size distribution &<br>Agglomeration/<br>Aggregation state**	DLS: (Correlation Func., Number Distribution)<br>101.3 nm +/-1.5 ; PDI= 0.115<br>TEM: d50= 78 nm, d90= 96 nm<br>AC: d50= 77 nm, d90= 100 nm
**Shape**	quasi spherical
**Crystal Structure**	cubic
**Surface Chemistry**	XPS: Atom% C 59.1 O 17.5, Ag 15.9, N 7.5<br>SIMS: Ag, Cl, $C_xH_yO_z$
**pH (in water)**	5.9
**Surface Charge**	Zeta potential: - 12.5 mV+/- 0.5<br>IEP: ~ pH 3.6

**Scanning (Raster) Electron Microscopy (SEM)**	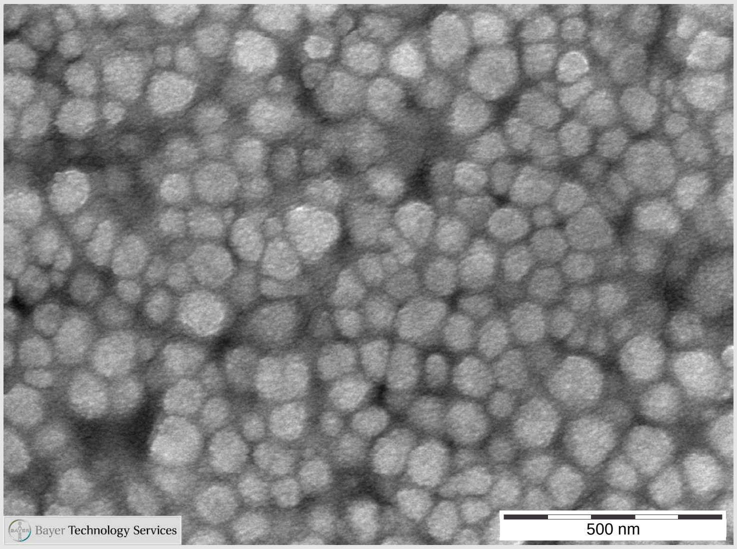
**Transmission Electron Microscopy (TEM)**	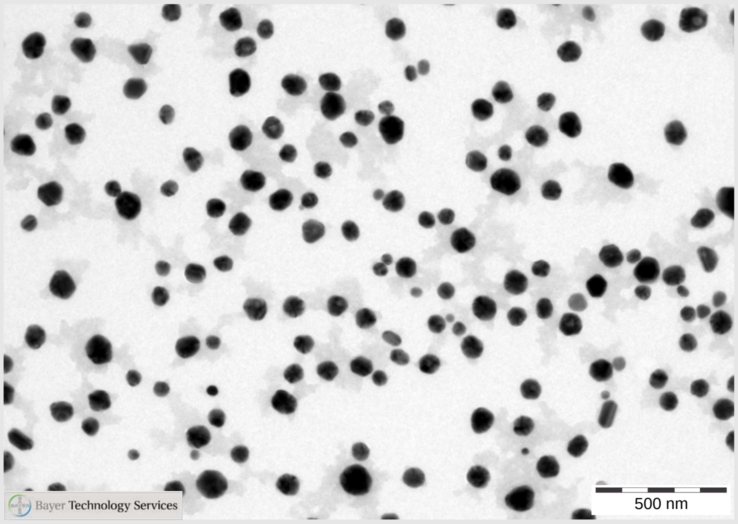<br>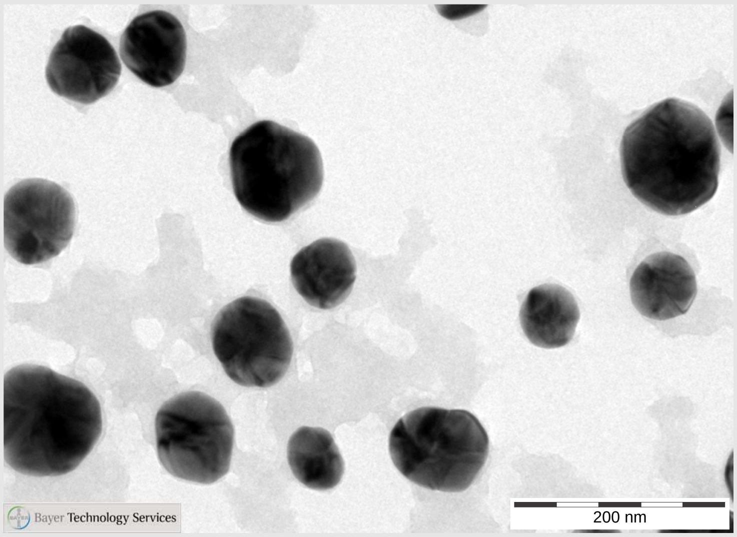

X-ray Diffraction (XRD)

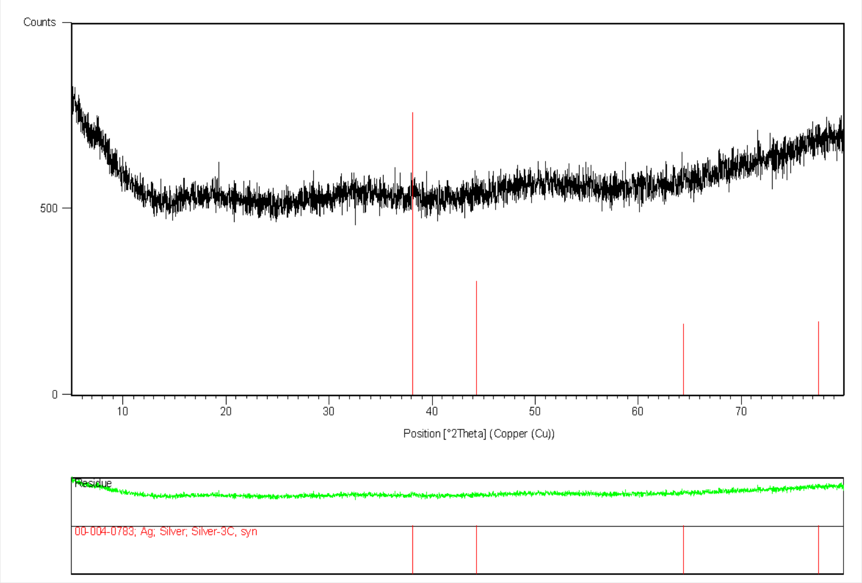

X-ray Photoelectron Spectroscopy (XPS)

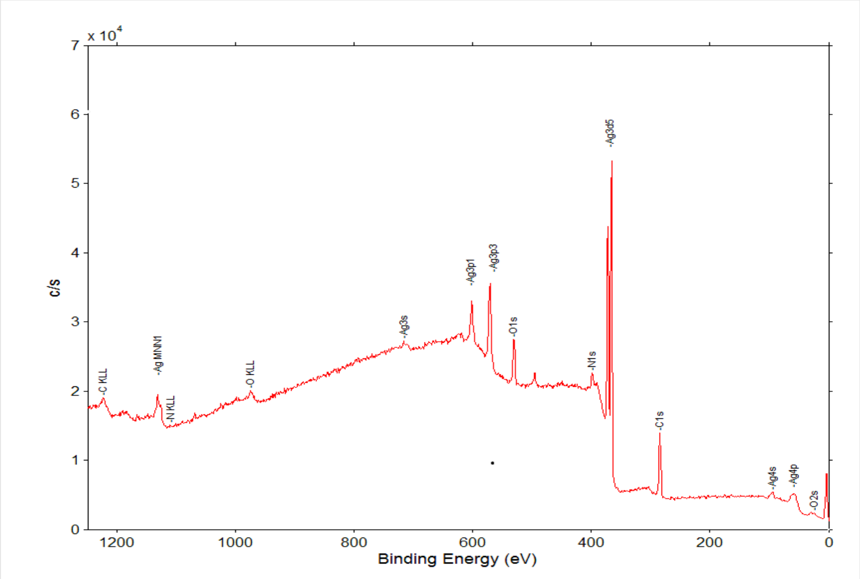

**Analytical Ultracentrifugation (AC)**	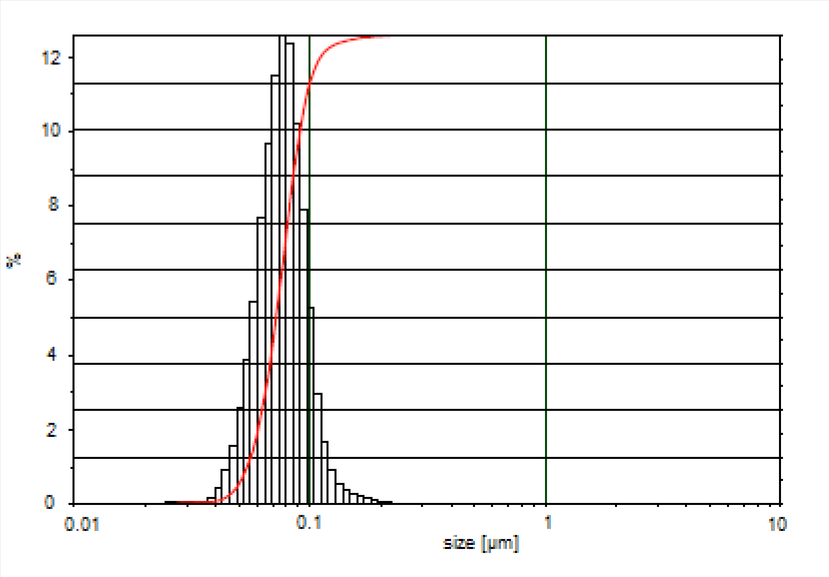
**Zeta Potential & Isoelectric Point**	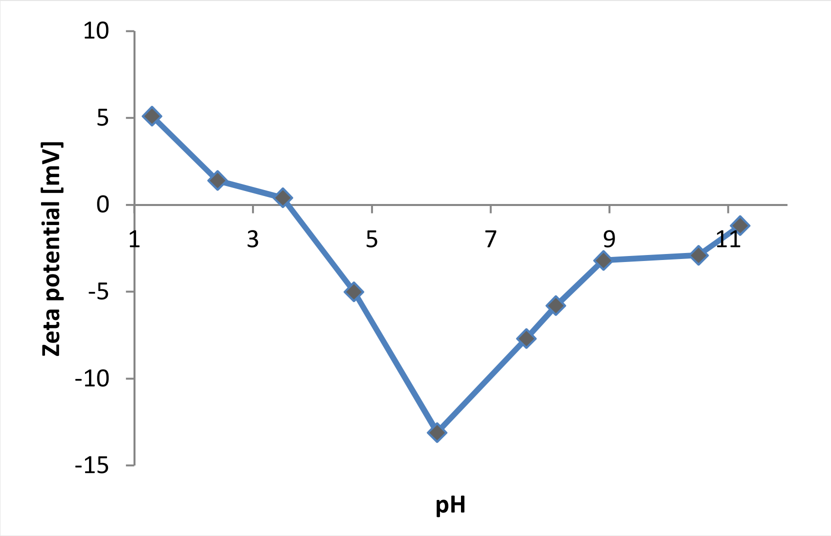
