# Supplementary material for: Is the toxic potential of nanosilver dependent on its size?
Source: Part Fibre Toxicol. 2014 Dec 3;11:65. doi: 10.1186/s12989-014-0065-1 (PMC4274708; doi:10.1186/s12989-014-0065-1)
Supplement: Additional file 3: Figure S3. — Detailed characterization of Ag ENMs 200 nm. [file 12989_2014_65_MOESM3_ESM.pdf]

NanoTOES
Nanotechnology: Training of Experts in Safety

Material Ag (200 nm)

**NanoTOES number**	NanoTOES_Ag_200
**Provider**	Bayer Technology Services GmbH
**Concentration**	1 % (wt/wt) in water<br>1.94*10^{12} NPs/ml
**Specific Surface Area**	3.87 m^2/g
**Size/<br>Size distribution &<br>Agglomeration/<br>Aggregation state**	DLS: (Correlation Func., Number Distribution)<br>272.5 nm +/-2.2 ; PDI= 0.136<br>TEM: d50= 168 nm, d90= 255 nm<br>AC: d50= 150 nm, d90= 380 nm
**Shape**	quasi spherical
**Crystal Structure**	cubic
**Surface Chemistry**	XPS: Atom % C 58.1, O 18.0, Ag 15.4, N 7.5, Na 1.0<br>SIMS: Ag, Na, K, $C_xH_yO_z$
**pH (in water)**	6.0
**Surface Charge**	Zeta potential: - 5.5 mV+/- 0.5<br>IEP: ~ 4.2 pH

**Scanning (Raster) Electron Microscopy (SEM)**	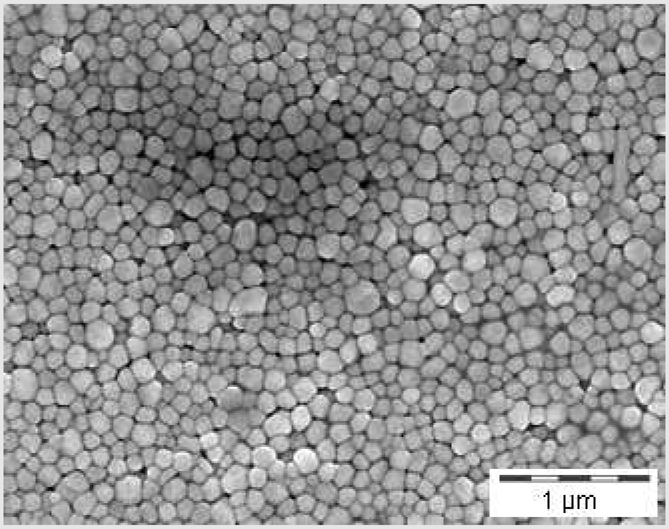
**Transmission Electron Microscopy (TEM)**	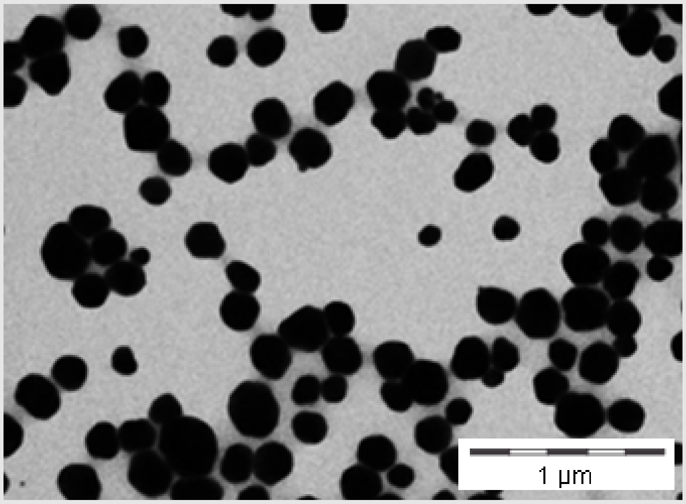 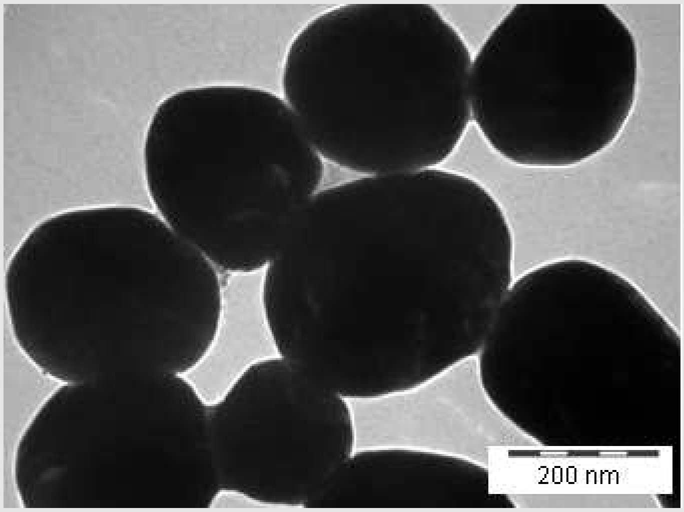

**X-ray Diffraction (XRD)**	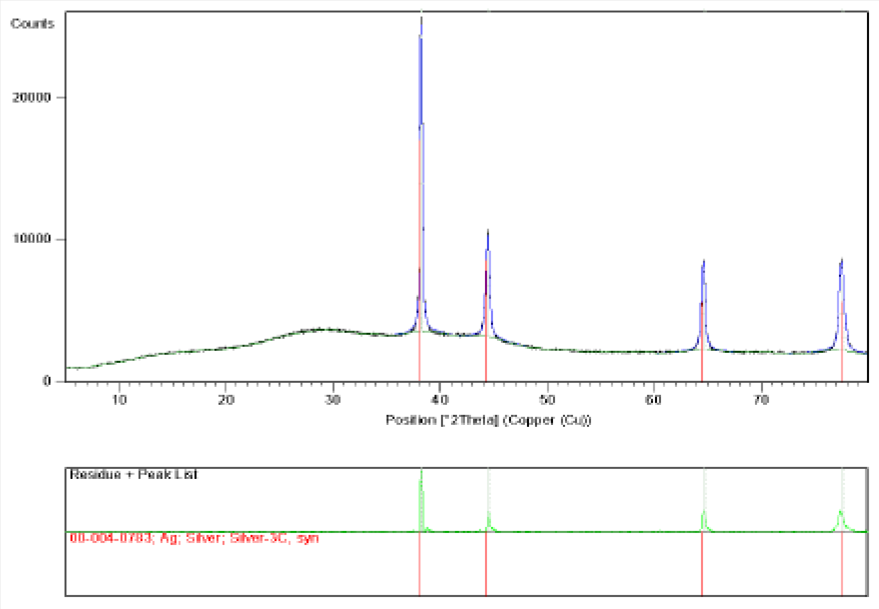
**X-ray Photoelectron Spectroscopy (XPS)**	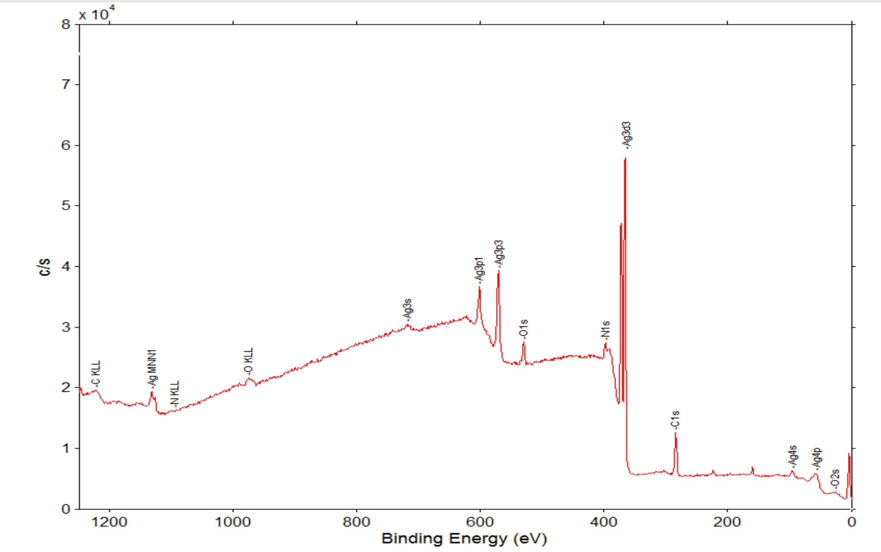

**Analytical Ultracentrifugation (AC)**	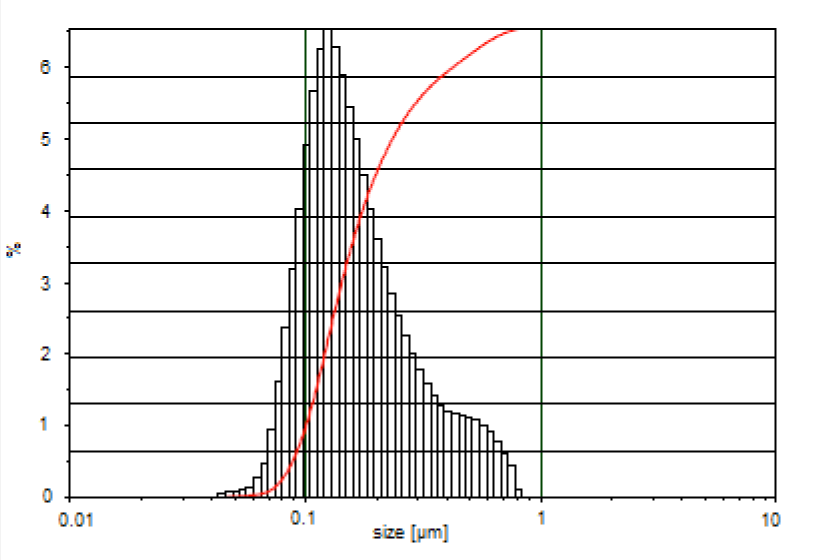
**Zeta Potential & Isoelectric Point**	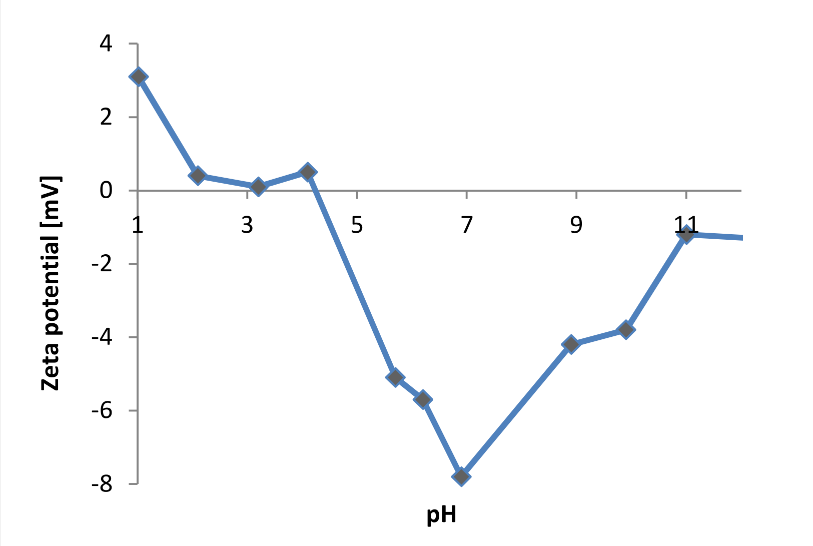
