# Supplementary material for: Is the toxic potential of nanosilver dependent on its size?
Source: Part Fibre Toxicol. 2014 Dec 3;11:65. doi: 10.1186/s12989-014-0065-1 (PMC4274708; doi:10.1186/s12989-014-0065-1)
Supplement: Additional file 4: Figure S4. — Light microscopy (100x) of A549 cells incubated with 50, 80, 200 nm Ag ENMs (concentration 21.2 μg/cm2) for 24 h. A: Negative control, B: Cells treated with Ag ENMs 50 nm; C: cells treated with Ag ENMs 80 nm; D: cells treated with Ag ENMs 200 nm. Figure S5. Cytotoxic effects of 50, 80 and 200 nm Ag ENMs on A549 cells measured as Relative growth activity (RGA) and Plating efficiency (PE). Concentrations of Ag ENMs expressed as ENMs cm2/cm2. Figure S6. Cytotoxic effects of 50, 80 and 200 nm Ag ENMs on A549 cells measured as Relative growth activity (RGA) and Plating efficiency (PE). Concentrations of Ag ENMs expressed as ENMs/cm2. Figure S7. Level of DNA damage – strand breaks and oxidised DNA lesions expressed as NET FPG in A549 cells exposed to different concentrations of Ag ENMs. Concentrations of Ag ENMs expressed as ENMs cm2/cm2. Figure S8. Level of DNA damage – strand breaks and oxidised DNA lesions expressed as NET FPG in A549 cells exposed to different concentrations of Ag ENMs. Concentrations of Ag ENMs expressed as ENMs/cm2. Figure S9. Induction of IL-8 and MCP-1 in A549 cells exposed to Ag ENMs 50, 80 and 200 nm. Concentrations of Ag ENMs expressed as ENMs cm2/cm2. Figure S10. Induction of IL-8 and MCP-1 in A549 cells exposed to Ag ENMs 50, 80 and 200 nm. Concentrations of Ag ENMs expressed as ENMs/cm2. Figure S11. Effect of 24 h treatment with 50, 80 and 200 nm Ag ENMs on induction of hprt gene mutations in V79-4 cells. Concentrations of Ag ENMs expressed as ENMs cm2/cm2. Figures S12. Effect of 24 h treatment with 50, 80 and 200 nm Ag ENMs on induction of hprt gene mutations in V79-4 cells. Concentrations of Ag ENMs expressed as ENMs/cm2. [file 12989_2014_65_MOESM4_ESM.docx]

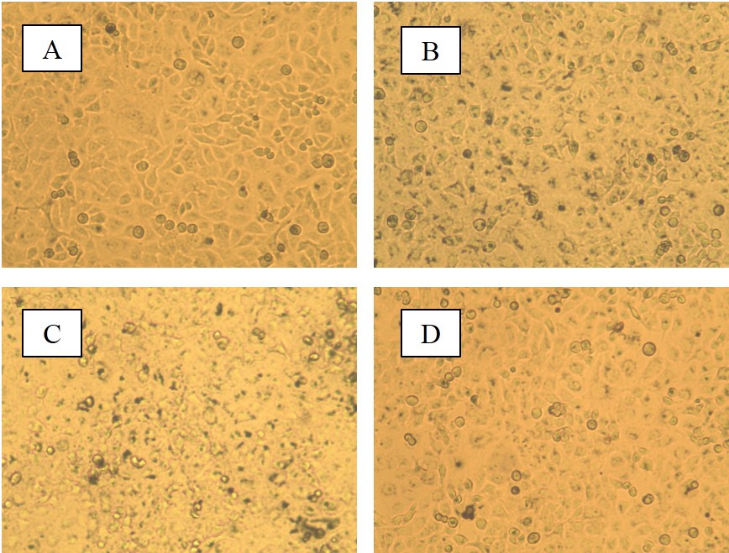


**Figure. S4:** Light microscopy (100x) of A549 cells incubated with 50, 80, 200 nm Ag ENMs (concentration 21.2 µg/cm^2^) for 24 h. A: Negative control, B: Cells treated with Ag ENMs 50 nm; C: cells treated with Ag ENMs 80 nm; D: cells treated with Ag ENMs 200 nm.

| 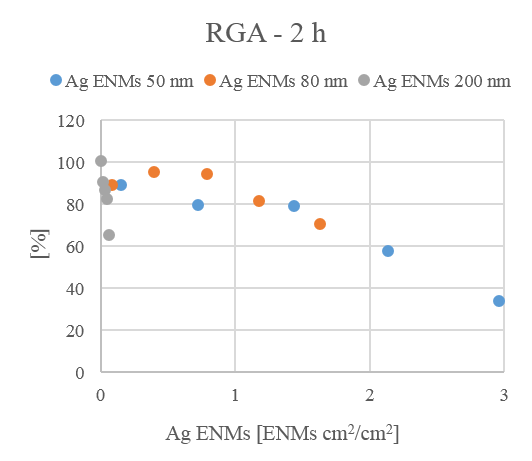 | 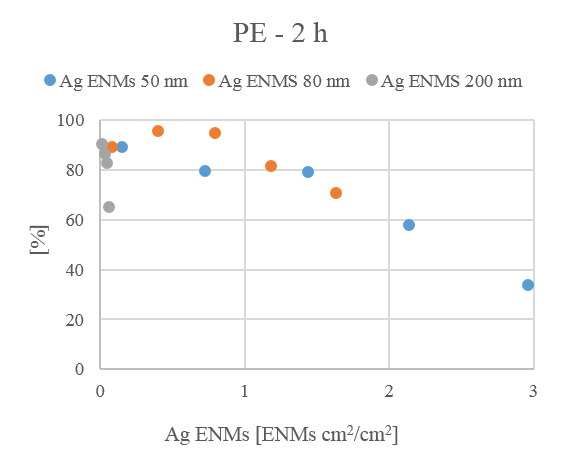 |
| --- | --- |
| 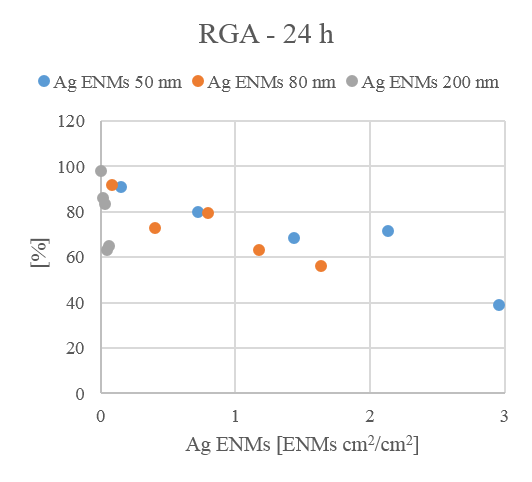 | 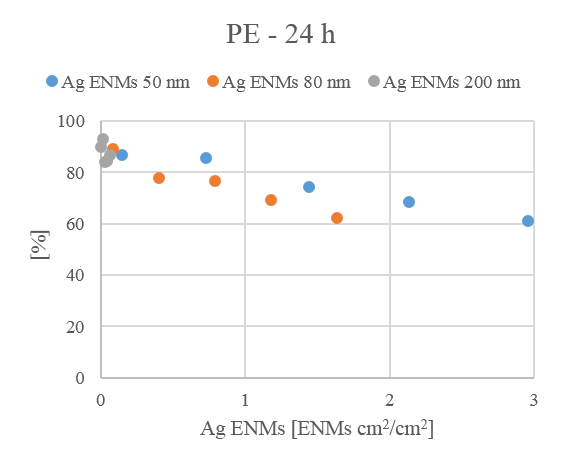 |
| 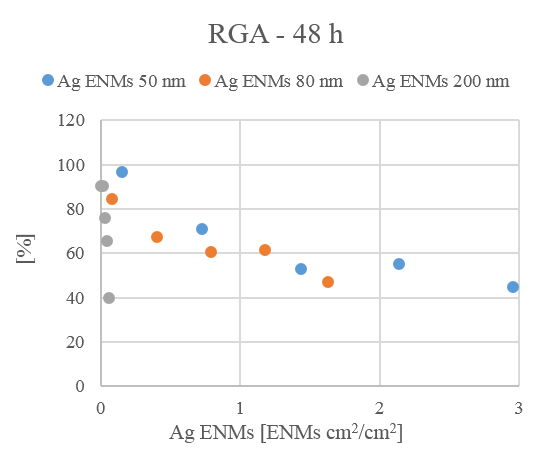 | 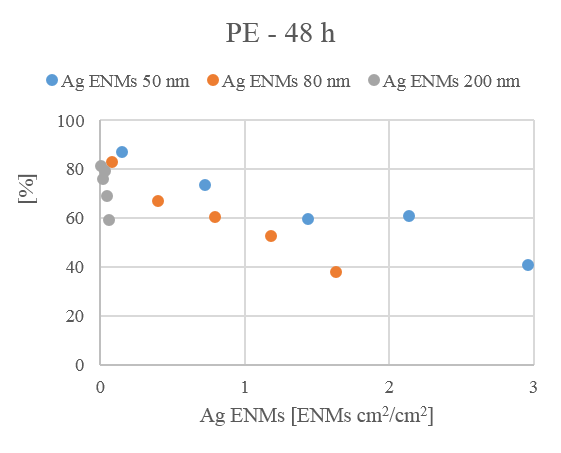 |

**Supplemental figure S5:** Cytotoxic effects of 50, 80 and 200 nm Ag ENMs on A549 cells measured as Relative growth activity (RGA) and Plating efficiency (PE). Ag ENMs concentrations are expressed in surface area of ENMs per cm^2^ [ENMs cm^2^/cm^2^]. Points represents cytotoxicity relative to 100 % of control. The data are expressed as mean of three independent experiments.

| 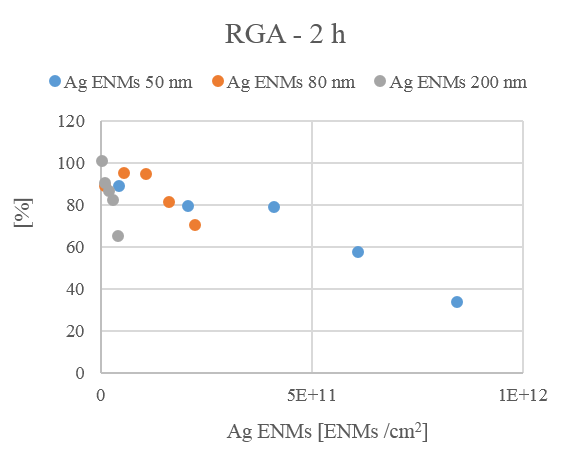 | 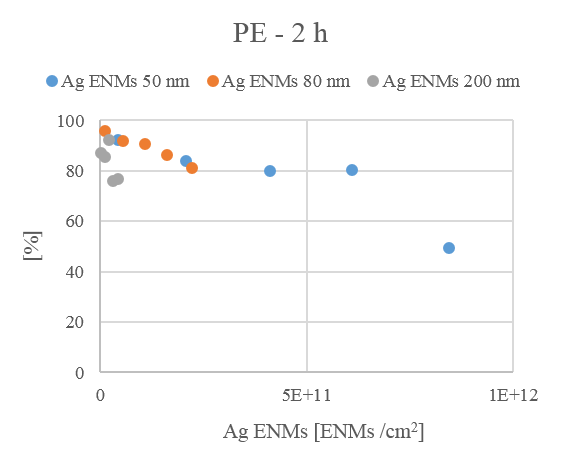 |
| --- | --- |
| 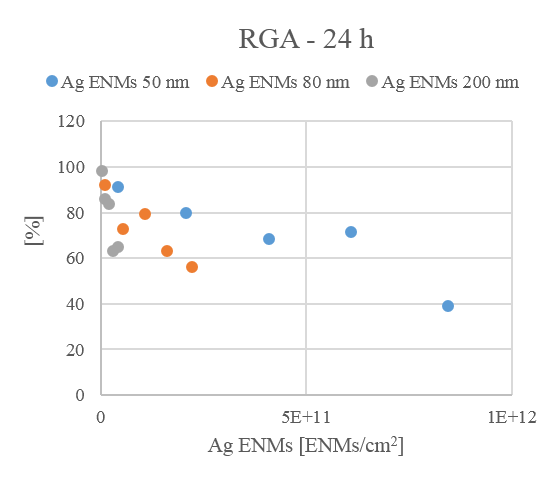 | 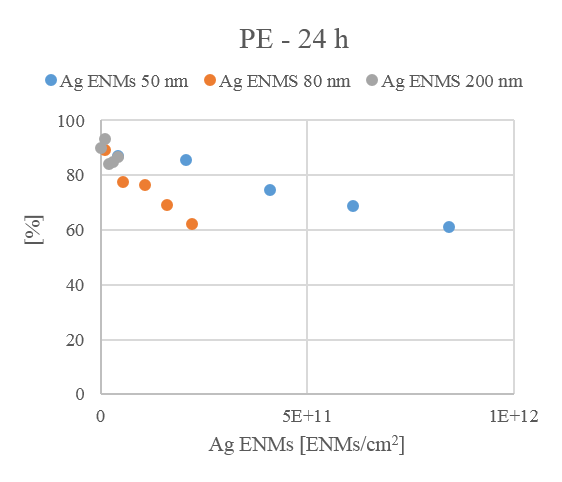 |
| 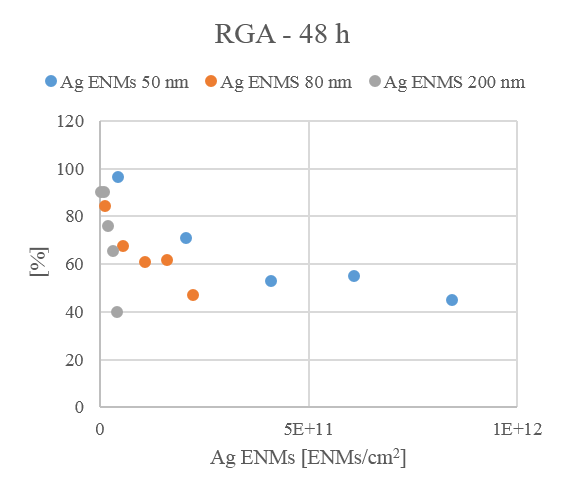 | 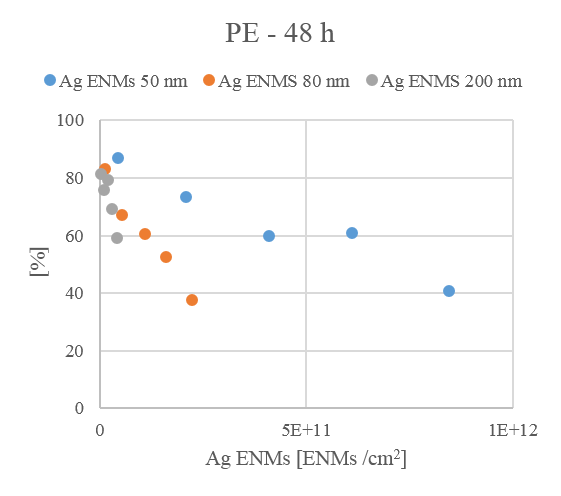 |

**Supplemental figure S6:** Cytotoxic effects of 50, 80 and 200 nm Ag ENMs on A549 cells measured as Relative growth activity (RGA) and Plating efficiency (PE). Ag ENMs concentrations are expressed in number of ENMs per cm^2^ [ENMs/cm^2^]. Points represents cytotoxicity relative to 100% of control. The data are expressed as mean of three independent experiments.

| 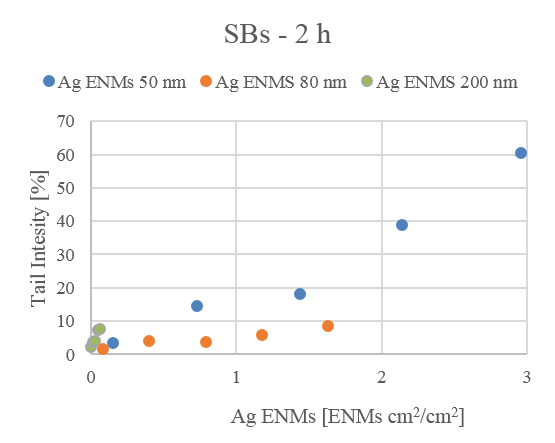 | 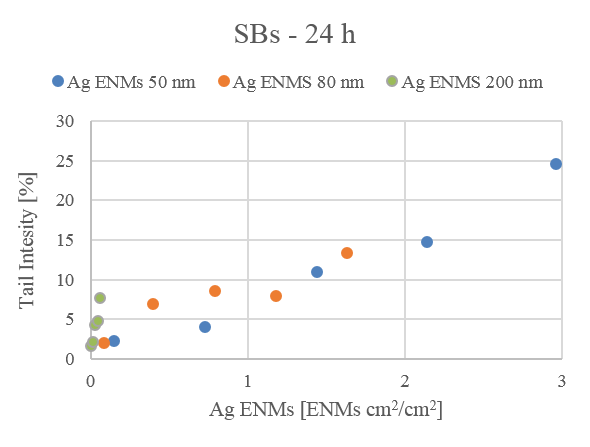 |
| --- | --- |
| 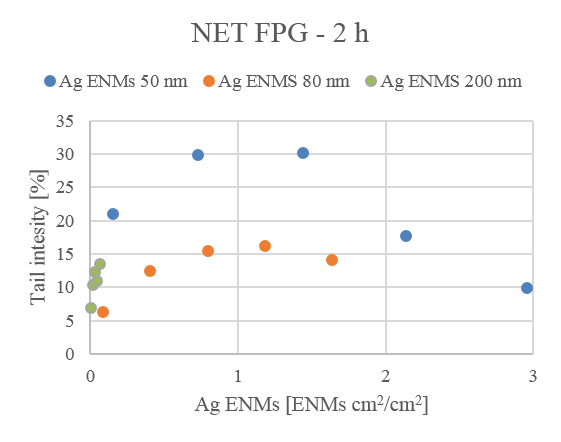 | 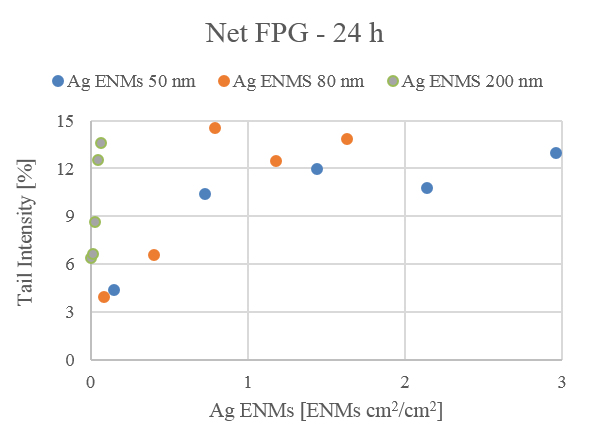 |

**Supplemental figure S7:** Level of DNA damage – strand breaks (SBs) and oxidised DNA lesions expressed as NET FPG in A549 cells exposed to different concentrations of Ag ENMs. Ag ENMs concentrations expressed in surface area of ENMs on cm^2^ [ENMs cm^2^/cm^2^].

| 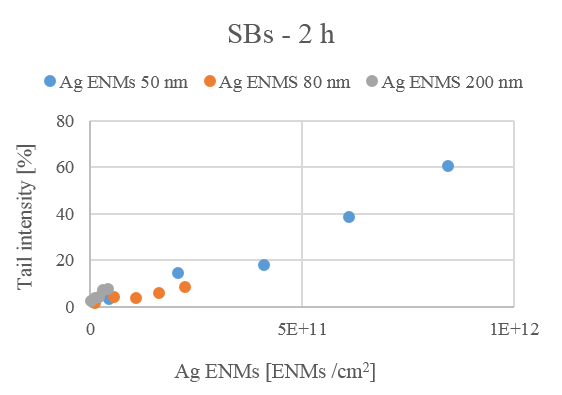 | 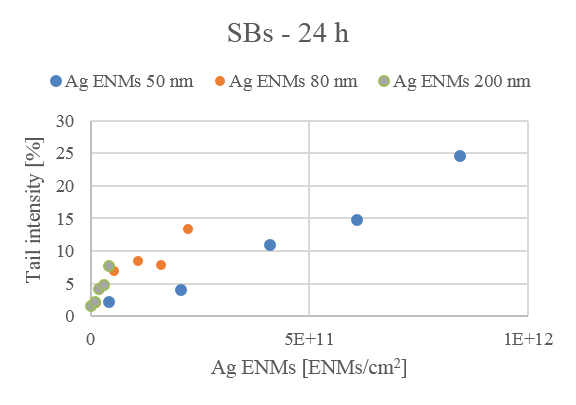 |
| --- | --- |
| 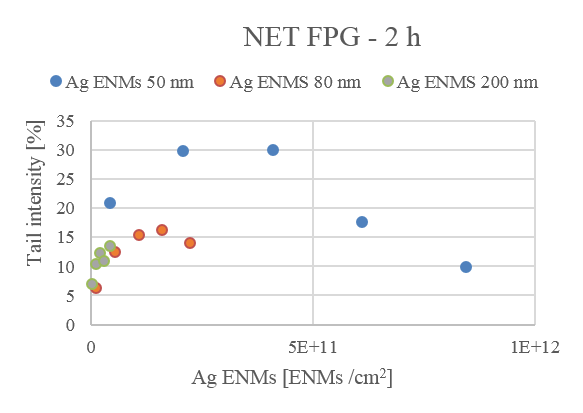 | 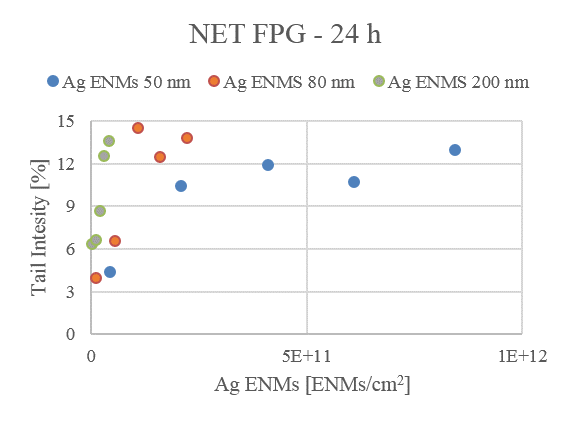 |

**Supplemental figure S8:** Level of DNA damage – strand breaks and oxidised DNA lesions expressed as NET FPG in A549 cells exposed to different concentrations of Ag ENMs. Ag ENMs concentrations are expressed in number of ENMs on cm^2^ [ENMs/cm^2^]. Ag ENMs concentrations expressed in number of ENMs on cm^2^ [ENMs cm^2^/cm^2^].

| 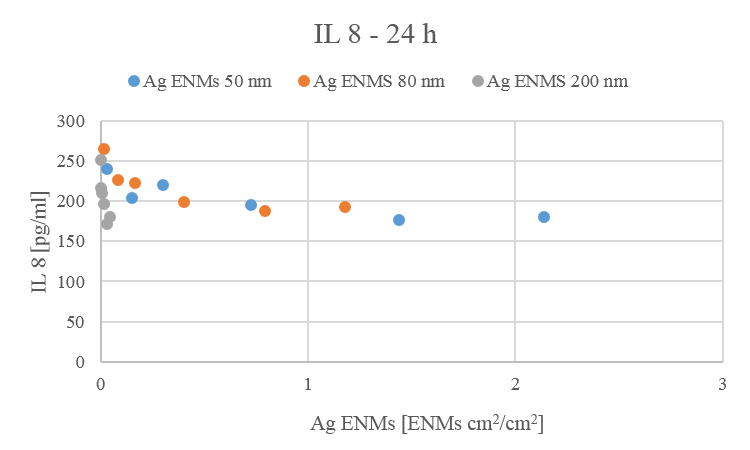 |
| --- |
| 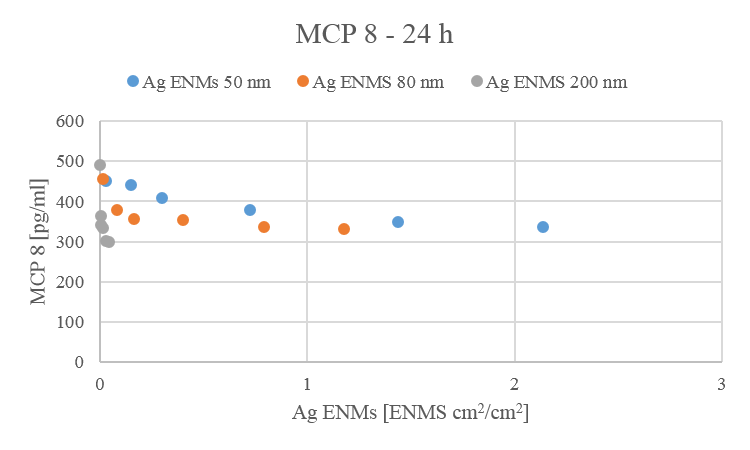 |

**Supplemental figure S9:** Induction of IL-8 and MCP-1 in A549 cells exposed to Ag ENMs 50, 80 and 200 nm. The data are expressed as mean of 4 independent experiments. Ag ENMs concentrations are expressed in surface area of ENMs on cm^2^ [ENMs cm^2^/cm^2^].

| 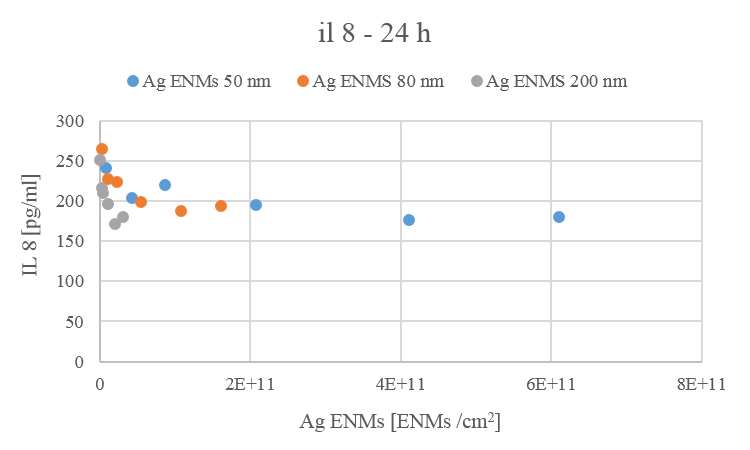 |
| --- |
| 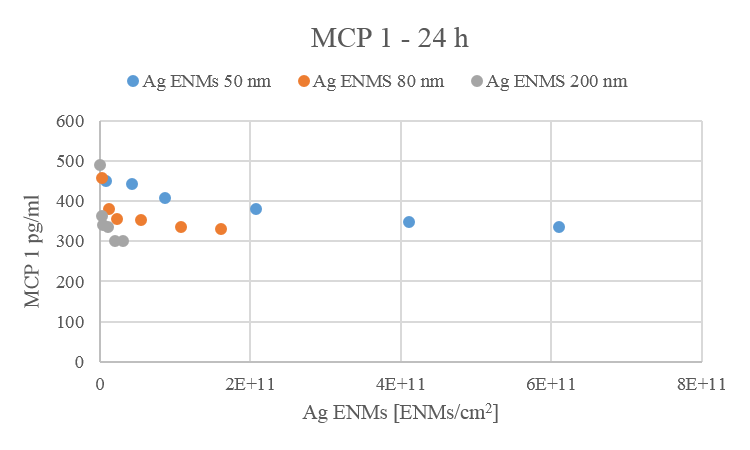 |

**Supplemental figure S10:** Induction of IL-8 and MCP-1 in A549 cells exposed to Ag ENMs 50, 80 and 200 nm. The data are expressed as mean of 4 independent experiments. Ag ENMs concentrations are expressed in number of ENMs on cm^2^ [ENMs/cm^2^].


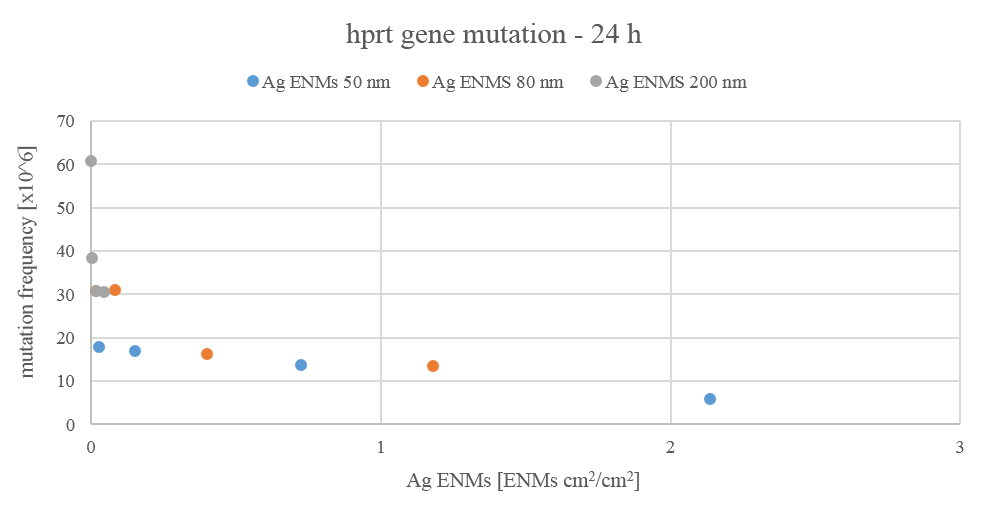


**Supplemental figure S11:** Effect of 24 h treatment with 50, 80 and 200 nm Ag ENMs on induction of *hprt* gene mutations in V79-4 cells. The mutant frequencies (×10^6^) are expressed as mean of two independent experiments, with two independent harvests per experiment. Ag ENMs concentrations are expressed in surface area of ENMs on cm^2^ [ENMs cm^2^/cm^2^].


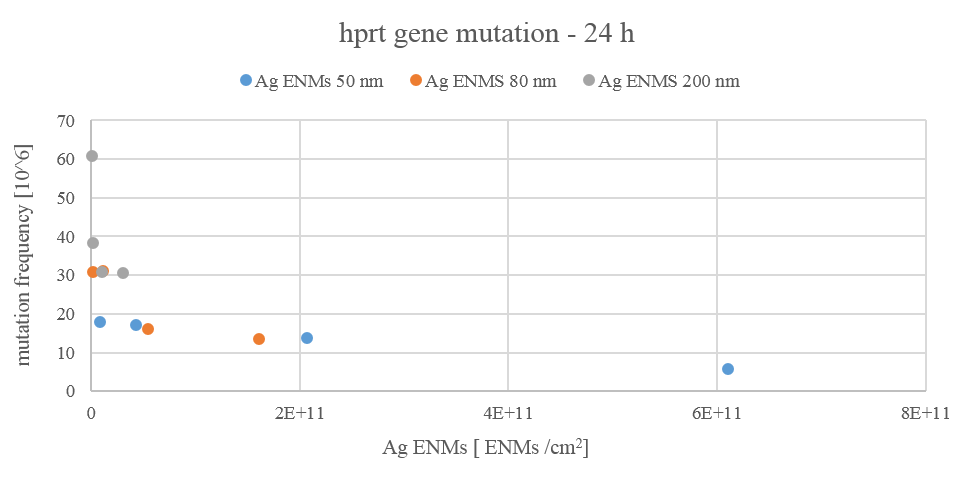


**Supplemental figure S12:** Effect of 24 h treatment with 50, 80 and 200 nm Ag ENMs on induction of *hprt* gene mutations in V79-4 cells. The mutant frequencies (×10^6^) are expressed as mean of two independent experiments, with two independent harvests per experiment. Ag ENMs concentrations are expressed in number of ENMs on cm^2^ [ENMs/cm^2^].
